# ZIP10 as a potential therapeutic target in acute myeloid leukaemia

**DOI:** 10.1111/bjh.20229

**Published:** 2025-06-30

**Authors:** Benjamin Rolles, Nicolas Chatain, Richard Görg, Margherita Vieri, Nora Tillmann‐Tröster, Marcel G. Bourgeois, Deborah Christen, Jeanette Walter, Addison C. Hillerbrand, Kyle A. Romine, Kathryn M. Taylor, Jens Bertram, Edgar Jost, Steffen Koschmieder, Fabian Beier, Maximilian Stahl, Tim H. Brümmendorf, Lothar Rink, Inga Wessels

**Affiliations:** ^1^ Department of Hematology, Oncology, Hemostaseology and Stem Cell Transplantation, Medical Faculty RWTH Aachen University Aachen Germany; ^2^ Center for Integrated Oncology Aachen Bonn Cologne Duesseldorf (CIO ABCD) Aachen Germany; ^3^ Institute of Immunology, Medical Faculty RWTH Aachen University Aachen Germany; ^4^ Division of Hematology, Department of Medicine, Brigham and Women's Hospital Harvard Medical School Boston Massachusetts USA; ^5^ Department of Medical Oncology Dana‐Farber Cancer Institute Boston Massachusetts USA; ^6^ Breast Cancer Molecular Pharmacology Unit, School of Pharmacy and Pharmaceutical Sciences Cardiff University Cardiff UK; ^7^ Institute for Occupational, Social and Environmental Medicine, Medical Faculty RWTH Aachen University Aachen Germany; ^8^ Center of Allergy and Environment Technical University of Munich, School of Medicine and Health & Helmholtz Munich Munich Germany

**Keywords:** acute myeloid leukaemia, AML, leukaemia, trace elements, zinc

## Abstract

Acute myeloid leukaemia (AML) is a haematopoietic malignancy that continues to demonstrate lapses in current treatment modalities as evidenced by therapy refractory disease, disease relapse and high rates of lethality. The influence of nutritional factors, including trace elements, on disease development and progression is not yet well understood. We utilized AML cell lines and patient samples to further investigate zinc homeostasis and the dependency of leukaemic cells on zinc. Compared to control individuals, we found significantly increased zinc levels in malignant blasts with concomitant serum hypozincaemia. Increased cellular zinc levels were accompanied by the upregulation of zinc influx transporters such as *ZIP6*, *ZIP9* and *ZIP10*. Subsequent in vitro experiments showed the importance of zinc for myeloid cell proliferation, survival and block of differentiation. We validated our results with data from the Leukemia Mile (*n* = 542) and the BeatAML2.0 study (*n* = 805). Importantly, we identified ZIP10 (as one of the highly upregulated zinc transporters in malignant blasts) which, when targeted, resulted in impaired zinc uptake and decreased malignant cell growth. These findings suggest that therapeutic approaches that target the zinc influx transporter ZIP10 may offer novel means of treatment for patients suffering from AML.

## Graphical abstract

Increased intracellular zinc levels in acute myeloid leukaemia blasts with upregulated expression of zinc influx transporters *ZIP6* and *ZIP10*. Targeting ZIP10 with a surface antibody represents a potential therapeutic strategy. Created in BioRender. Rolles, B. (2025) https://BioRender.com/bwrtof1.

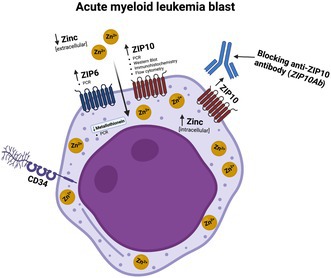

## INTRODUCTION

Despite advancements in understanding myeloid malignancies, effectively treating patients with acute myeloid leukaemia (AML) remains a major challenge. Currently, about 40%–50% of all AML patients below 60 years of age relapse after first‐line therapy. Second‐/third‐line therapies are associated with significantly worse treatment responses.^1^ In particular, patients with high‐risk mutations, secondary AML and blast phase myeloproliferative neoplasms (MPNs) show a poor prognosis.^2^, ^3^ The standard treatment for patients with intermediate‐ or high‐risk AML is an allogenic stem cell transplantation (allo‐SCT). Nevertheless, about 20%–50% of all transplanted patients relapse, illustrating the strong need for improved therapies.^4^ Nonetheless, how leukaemic stem cells can survive after allo‐SCT is not sufficiently understood so far.

The benefits of zinc on immune cell function has been intensively studied demonstrating its importance for cell homeostasis, cell survival and proliferation.^5^, ^6^ Zinc is an important enzymatic cofactor and impacts the function of at least 300 different human enzymes. As a second messenger, zinc is crucial for genetic stability and for the functioning of various signalling cascades.^6^, ^7^ Clinically apparent zinc deficiency mainly manifests itself in body tissues with high cell turnover.^5^ Thus, patients with zinc deficiency present with hair loss, mucosal lesions, eczematous skin alterations, wound healing disorders, diarrhoea and impairment of the immune system.^6^, ^8^ Interestingly, a recent study showed that the efficiency of allo‐SCT with concomitant T‐cell reconstitution can be improved by supplementation with the trace element zinc thereby arguing for its administration.^9^, ^10^

Due to its vital role on cell proliferation, we hypothesize that increased zinc supply may also have adverse effects on AML pathogenesis and disease‐free survival after allo‐SCT.^9^ While intrinsic mechanisms of AML pathogenesis, such as mutations and epigenetic changes, are well investigated, research focusing on the impact of vitamins and trace elements is underrepresented. Some recent papers already discuss zinc transporters as a potential therapeutic target for various diseases^11^ and show low zinc levels in AML patients,^12^ but detailed studies are lacking. Interestingly, a recent study showed a strong dependency between AML blasts and vitamin B6 levels indicating the importance of nutritional factors.^13^ Furthermore, as early as the 1940s, changes in the folic acid metabolism in children with acute lymphoid leukaemia (ALL), uncovered by Sidney Farber, led to the development of a new class of drugs, the folic acid antagonists, which revolutionized ALL therapy.^14^

Despite strong indications that altered zinc homeostasis may impact AML pathogenesis, work in this regard is rare. Therefore, we investigated the question of whether zinc has supportive or detrimental effects on a malignant myeloid disease such as AML by using various cell lines, primary patient samples and publicly available datasets.

## METHODS

The methods used are described in the Appendix S1.

## RESULTS

### Intracellular zinc levels are increased in peripheral blood and bone marrow of AML patients

**FIGURE 1:**
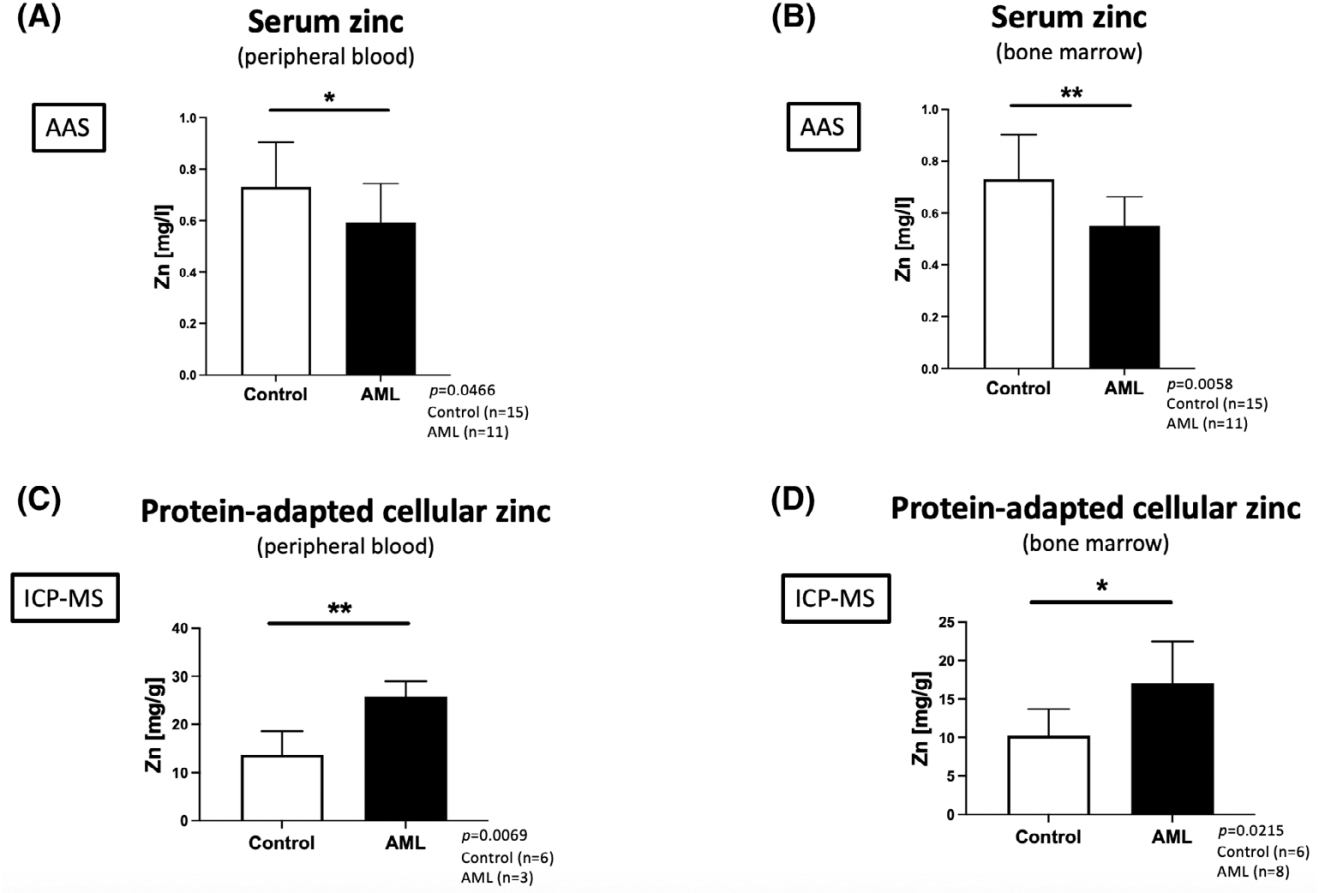
Zinc level in patients with first diagnosis or relapse of acute myeloid leukaemia. Atomic absorption spectroscopy (AAS) was used to measure the zinc concentration in (A) blood and (B) bone marrow serum (control subjects: *n* = 15, patients with first diagnosis of acute myeloid leukaemia (AML): *n* = 11). The protein‐adapted cellular zinc concentration was measured after cell lysis by using inductively coupled plasma mass spectrometry (ICP‐MS) in (C) peripheral blood cells (control subjects: *n* = 6, AML: *N* = 3) and (D) bone marrow cells (control subjects: *n* = 6, AML: *n* = 8). The control cohort consisted of patients with a diagnosis other than leukaemia without bone marrow and/or blood involvement (*n* = 5), patients cured after successful AML therapy (*n* = 3) and patients with AML after successful allogenic stem cell transplantation (*n* = 7). Data are displayed as mean + standard deviation (SD). Significance is indicated by *p < 0.05 and **p < 0.01 (Student's *t*‐test).

First, we used patient biomaterial to evaluate serum zinc levels in the peripheral blood (PB) (Figure 1A, *n* = 11) and bone marrow (BM) (Figure 1B, *n* = 11). Biomaterial from patients (serum zinc: *n* = 15; total cellular zinc: *n* = 6) who had either been cured from AML by chemotherapy alone (*n* = 3), recovered from AML after successful allo‐SCT (*n* = 7) or had another (malignant) diagnosis without BM involvement (*n* = 5) served as controls. All AML patients had a blast infiltration of at least 23% in the BM. Samples used to measure the total cellular zinc amount in cells from the PB had a minimum peripheral blast count of 17%. Characteristics of all recruited AML patients (*n* = 43) including co‐mutations at the time of diagnosis and overall survival are shown in Table 1 and Table S1. Zinc concentrations in the serum of PB (Figure 1A; *p* = 0.0466) and BM (Figure 1B; *p* = 0.0058) were significantly lower in patients with AML compared to control individuals. Next, in cases where sufficient leucocyte numbers were isolated, total cellular zinc was assessed for PB (*n* = 3) and BM (*n* = 8) cells (Figure 1C,D). We found that intracellular zinc levels were significantly higher in PB cells (Figure 1C; *p* = 0.0069) and BM cells (Figure 1D; *p* = 0.0215) from patients with first diagnosis of AML compared to control subjects. Copper levels and the zinc/copper ratio were not significantly altered in patients with AML compared to control subjects (Figure S1).

**TABLE 1:** Clinical information. [Colour table can be viewed at wileyonlinelibrary.com]

Sample No.	Gender	Age at time of sampling	Diagnosis	% blasts (PB)	Biomaterial (PB)	% blasts (BM)	Biomaterial (BM)	FLT3‐ITD	FLT3‐TKD	NPM1	ASXL1	RUNX1	IDH1	IDH2	CEBPA	TP53	DNMT3A	TET2	KMT2A	Survival	Days of survival
AML No. 1	F	65	First diagnosis AML (Fab M4)	85	Yes	73	Yes	+	−	+	−	−	−	−	−	−	+	−	−	No	300
AML No. 2	F	75	First diagnosis AML (Fab M1)	52	Yes	80	Yes	+	−	+	−	−	−	−	−	−	−	−	−	No	24
AML No. 3	M	68	First diagnosis AML (Fab M4)	52	Yes	82	Yes	−	−	+	−	−	−	−	−	−	−	−	−	Yes	N/A
AML No. 4	F	47	First diagnosis AML (Fab M4)	17	Yes	52	Yes	−	−	−	−	+	−	−	−	−	−	−	−	Yes	N/A
AML No. 5	F	78	Relapsed AML (Fab M2)	3	No	21	Yes	−	−	−	−	+	−	−	−	−	−	−	−	No	27
AML No. 6	F	71	Secondary AML (myelodysplastic syndrome)	40	Yes	32	Yes	−	−	−	+	−	−	+	−	−	−	−	−	Yes	N/A
AML No. 7	F	73	First diagnosis AML (Fab M5)	1	No	25	Yes	−	−	−	+	−	−	−	−	−	−	+	+	Yes	N/A
AML No. 8	F	62	Therapy‐related AML (breast cancer)	0	No	23	Yes	−	−	−	−	−	−	−	−	+	−	−	−	No	179
AML No. 9	M	50	First diagnosis AML (Fab unknown)	87	Yes	80	Yes	−	−	+	−	−	−	+	−	−	−	−	−	Yes	N/A
AML No. 10	F	71	Therapy‐refractory AML (Fab M1/2)	28	Yes	52	Yes	−	−	−	−	−	−	−	−	+	−	−	−	Yes	N/A
AML No. 11	F	67	First diagnosis AML (Fab unknown)	8	No	39	Yes	−	−	−	−	−	−	+	−	−	−	−	−	Yes	N/A
AML No. 12	M	58	First diagnosis AML (Fab M5)	73	Yes	91	Yes	−	−	+	−	−	−	−	−	−	−	−	−	Yes	N/A
AML No. 13	M	66	First diagnosis AML with myelodysplasia‐related changes	76	Yes	62	Yes	−	−	−	−	−	−	+	−	+	−	+	−	Yes	N/A
AML No. 14	M	75	Secondary AML (myelodysplastic syndrome)	8	No	36	Yes	/	−	−	−	−	−	−	/	−	+	−	−	Yes	N/A
AML No. 15	F	50	First diagnosis AML (Fab M4)	52	Yes	90	Yes	+	−	+	−	−	−	+	−	−	+	−	−	Yes	N/A
AML No. 16	M	52	First diagnosis AML (Fab unknown)	8	No	92	Yes	−	−	−	−	+	+	−	−	−	+	−	−	Yes	N/A
AML No. 17	F	52	Relapsed AML (Fab M1)	57	Yes	90	Yes	−	+	−	−	+	−	+	−	−	/	/	−	Yes	N/A
AML No. 18	M	46	First diagnosis AML (Fab M4)	47	Yes	48	Yes	+	+	−	−	−	−	−	−	−	+	−	−	Yes	N/A
AML No. 19	M	60	First diagnosis AML (Fab M4Eo)	23	Yes	52	Yes	−	−	−	−	−	−	−	−	−	−	−	−	Yes	N/A
AML No. 20	M	72	Secondary AML (SM‐AHN and myelodysplastic syndrome)	47	Yes	60	No	−	−	−	−	+	/	/	−	−	−	+	+	Yes	N/A
AML No. 21	M	64	First diagnosis AML (Fab M1)	80	Yes	84	No	+	−	+	−	−	−	−	−	−	−	−	−	Yes	N/A
AML No. 22	M	77	First diagnosis AML with myelodysplasia‐related changes	88	Yes	73	No	−	−	−	−	−	−	−	−	+	−	+	−	No	58
AML No. 23	M	63	Secondary AML (multiple myeloma)	48	Yes	80	Yes	−	+	−	−	−	−	−	+	+ +	+	−	−	No	9
AML No. 24	M	57	First diagnosis AML (Fab unknown)	25	Yes	80	No	−	−	−	+	−	−	−	−	−	−	+	−	No	22
AML No. 25	M	69	First diagnosis AML (Fab M4)	10	No	75	Yes	+	−	−	−	+	−	−	−	−	−	−	+	Yes	N/A
AML No. 26	F	78	First diagnosis AML (Fab M4)	26	No	48	Yes	−	−	−	−	+	/	/	−	+	/	/	−	Yes	N/A
AML No. 27	M	71	First diagnosis AML (Fab M4/5)	8	No	29	Yes	−	−	−	−	−	/	/	−	−	/	/	−	No	16
AML No. 28	F	65	First diagnosis AML with myelodysplasia‐related changes	71	Yes	32	No	−	−	−	−	+	−	+	−	−	−	−	−	Yes	N/A
AML No. 29	F	53	First diagnosis AML (Fab unknown)	87	Yes	82	Yes	−	−	+	−	−	−	−	−	−	+	−	−	Yes	N/A
AML No. 30	M	71	Therapy‐related AML (NSCLC)	59	Yes	87	No	−	−	−	/	−	/	/	−	+	/	/	−	No	19
AML No. 31	M	63	First diagnosis AML (Fab M5)	2	No	33	Yes	−	−	−	−	−	/	/	−	+	/	+	−	No	85
AML No. 32	M	80	Secondary AML (primary myelofibrosis)	18	Yes	N/A	No	−	−	−	/	−	/	/	−	/	/	/	−	Yes	N/A
AML No. 33	M	43	First diagnosis AML (Fab M1)	28	Yes	55	No	−	−	−	+	−	−	−	−	−	−	−	−	Yes	N/A
AML No. 34	F	62	First diagnosis AML with myelodysplasia‐related changes	48	No	65	Yes	−	−	−	−	−	−	+	−	−	+	−	−	Yes	N/A
AML No. 35	M	29	First diagnosis AML (Fab M4)	55	No	60	Yes	−	−	−	/	−	/	/	+	/	/	/	−	Yes	N/A
AML No. 36	F	75	Secondary AML (myelodysplastic syndrome)	8	No	60	Yes	−	−	−	−	−	−	−	−	−	+	−	−	Yes	N/A
AML No. 37	M	75	First diagnosis AML (Fab M1)	2	No	41	Yes	−	−	−	−	−	−	−	−	−	/	/	−	Yes	N/A
AML No. 38	F	76	First diagnosis AML with myelodysplasia‐related changes	1	No	30	Yes	−	−	−	−	+	−	−	−	+	−	+	−	No	334
AML No. 39	F	67	Therapy‐related AML (breast cancer)	86	No	84	Yes	−	−	−	−	−	−	−	−	−	−	−	+	Yes	N/A
AML No. 40	M	51	First diagnosis AML (Fab M1)	49	Yes	75	No	−	−	−	−	+	/	/	−	−	/	/	−	Yes	N/A
AML No. 41	F	54	Relapsed AML after HSCT	87	Yes	93	No	−	+	−	−	+	−	+	−	−	+	−	−	Yes	N/A
AML No. 42	M	78	Relapsed AML	65	Yes	70	No	−	−	−	+	+	−	−	−	−	−	−	+	Yes	N/A
AML No. 43	M	77	First diagnosis AML with myelodysplasia‐related changes	88	Yes	73	No	−	−	−	−	−	−	−	−	+	−	+	−	No	58

*Note*: Patient characteristics of all recruited AML patients. Patient material from the first patients were primarily used for initial zinc measurements. ‘+’: positive/present; ‘−’: negative/not present; ‘/’: not tested/unknown.

### Zinc influx transporters are upregulated in AML

**FIGURE 2:**
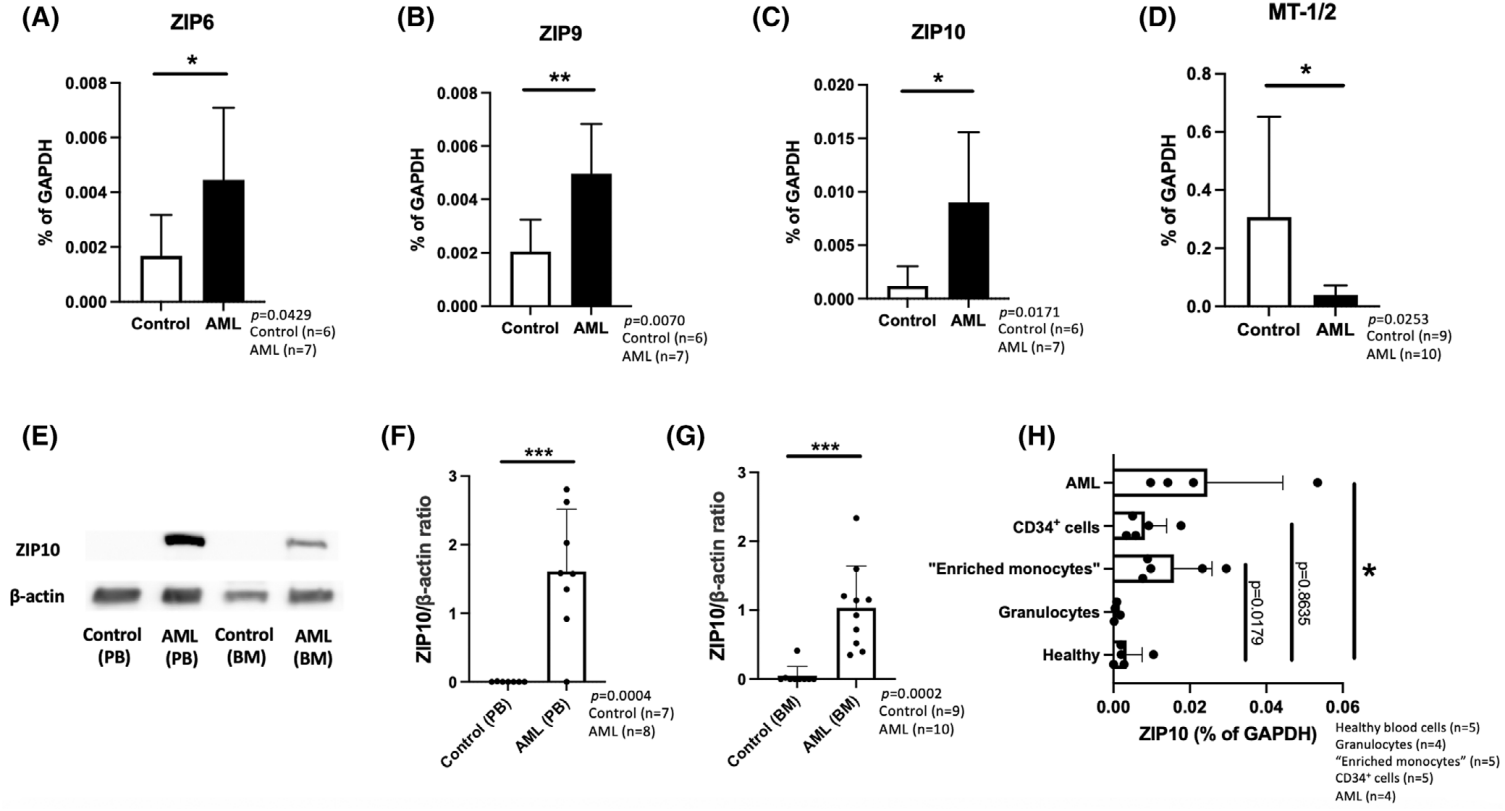
Zinc transporter expression in acute myeloid leukaemia. mRNA expression of (A) *Zrt‐ and Irt‐like protein (ZIP)6* (Control: *n* = 6, AML: *n* = 7; *p* = 0.0429), (B) *ZIP9* (Control: *n* = 6, AML: *n* = 7; *p* = 0.0070), (C) *ZIP10* (Control: *n* = 6, AML: *n* = 7; *p* = 0.0171) and (D) *metallothionein‐1/metallothionein‐2* (*MT‐1/2*) (Control: *n* = 9, AML: *n* = 10; *p* = 0.0253) were analysed in bone marrow cells after isolation with hydroxyethyl starch (HES) in control probands as well as patients with initial diagnosis of acute myeloid leukaemia (AML). Western blot analysis using (E + F) peripheral blood cells (PB) (control: *n* = 7, AML: *n* = 8; *p* = 0.0004) or (E + G) bone marrow cells (BM) (control: *n* = 9, AML: *n* = 10; *p* = 0.0002) from control individuals or patients with first diagnosis of AML after HES isolation. (H) *ZIP10* mRNA expression compared to healthy subjects (*n* = 5) in HES‐isolated peripheral blood cells of patients with first diagnosis of AML (*n* = 4; *p* = 0.0179), granulocytes isolated from healthy probands (*n* = 4; *p* = 0.9847), isolated (adherent) peripheral blood cells from healthy subjects (‘enriched monocytes’) (*n* = 5; *p* = 0.1890) and granulocyte‐colony‐stimulating factor (G‐CSF)‐mobilized CD34^+^ cells from healthy individuals (*n* = 5; *p* = 0.8635). (A–D + F + G): Student's *t*‐test and (H): One‐way ANOVA—Dunnett's multiple comparison were used to calculate statistical significance. Data are displayed as mean + standard deviation (SD). Significance is indicated by *p < 0.05, **p < 0.01 and ***p < 0.001 (Student's *t*‐test or one‐way ANOVA—Dunnett's multiple comparison).

To explain higher zinc concentrations in cells from AML patients, we screened the mRNA expression of *Zrt‐, Irt‐like proteins* (*ZIPs*) and *zinc transporters* (*ZnTs*) in the BM of patients with first‐diagnosis or relapsed AML. *ZIP1–ZIP14* (zinc influx transporters) and *ZnT1–ZnT10* (zinc efflux transporters) were analysed (Figure 2; Figure S2; Table S2). We found significantly higher expression of *ZIP6* (Figure 2A; *p* = 0.0429), *ZIP9* (Figure 2B; *p* = 0.0070) and *ZIP10* (Figure 2C; *p* = 0.0171) in treatment‐naïve AML patients. All three transporters are known to be responsible for zinc uptake into the cytoplasm. Interestingly, zinc storage proteins such as *metallothionein 1 and 2* (*MT‐1/2*) were less expressed in BM cells from AML patients compared to control samples (Figure 2D; *p* = 0.0253). Based on the mRNA expression results from all individual samples, the most significant differences between AML and control samples were observed for *ZIP10* and *MT‐1/2* (Figure S3A–D).

Therefore, we used available data from the Leukemia Mile Study^15^, ^16^, ^17^ and the BeatAML2.0 study^18^, ^19^ to confirm our data on increased *ZIP10* (=*SLC39A10*) expression in AML patients. Indeed, study data showed increased *ZIP10* expression in all AML subtypes including AML with normal karyotype (*n* = 351), complex karyotype (*n* = 48), inversion of chromosome 16 AML inv(16) (*n* = 28), acute promyelocytic leukaemia (AML FAB M3) with PML‐RARα translocation (AML t(15;17); *n* = 37), translocation of chromosome 8 and 21 (AML t(8;21)) (*n* = 40) and KMT2A (MLL)‐rearrangement (AML (MLL)) (*n* = 38) compared to healthy controls (*n* = 73) (Figure S3E). On the other hand, *ZIP10* mRNA expression in patients with myelodysplastic neoplasms (MDS) (*n* = 206)—a disease that predisposes individuals to the development of AML—was not increased compared to healthy subjects (*n* = 73) (Figure S3F). We also confirmed an increase of *ZIP10* in PB (*p* = 0.0004) and BM (*p* = 0.0002) from AML patients on protein level by western blotting (Figure 2E–G).

Interestingly, we found cell type specific differences in *ZIP10* mRNA expression by comparing those to peripheral blood mononuclear cells (PBMCs) from healthy donors (‘Healthy’). Leucocytes (*n* = 5) and granulocytes (*n* = 4) isolated from healthy donors showed low *ZIP10* mRNA expression (Figure 2H). In contrast, enriched monocytes (*n* = 5) exhibited higher *ZIP10* mRNA expression compared to PBMCs from healthy donors (*n* = 5, *p* = 0.1890). A similar tendency was observed in granulocyte colony stimulating factor (G‐CSF)‐mobilized CD34^+^ cells from healthy individuals (*n* = 5, *p* = 0.8635) (Figure 2H).

### Deleterious effects of zinc deprivation on AML cell lines

**FIGURE 3:**
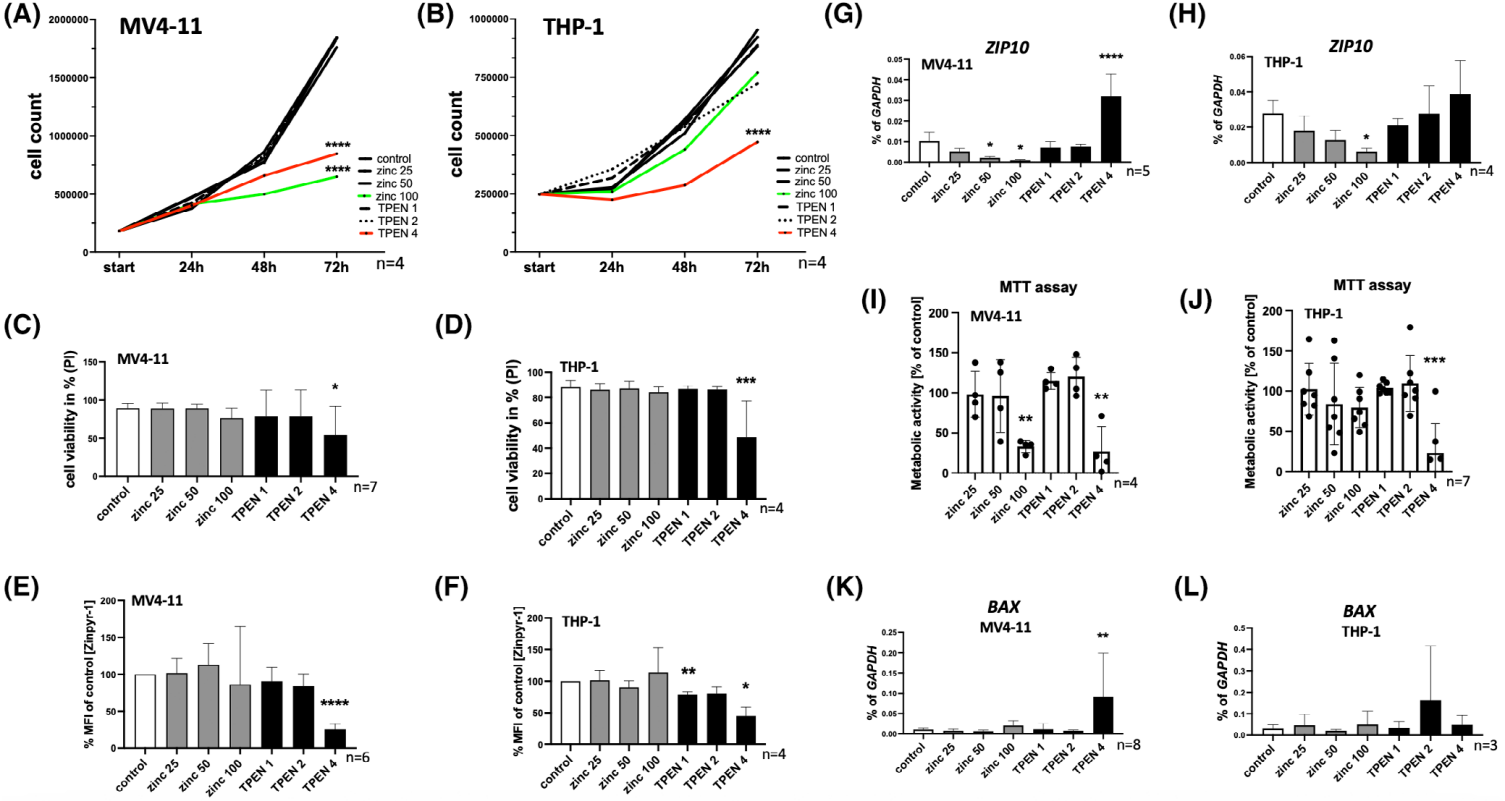
Deleterious effects of zinc deprivation on AML cell lines. Cell growth of (A) MV4‐11 (*n* = 4) and (B) THP‐1 cells (*n* = 4) under zinc‐rich (25 μM zinc sulphate, 50 μM zinc sulphate, 100 μM zinc sulphate) and zinc‐depleted conditions by using N,N,N′,N′‐tetrakis(2‐pyridinylmethyl)‐1,2‐ethanediamine (TPEN) (1 μM TPEN, 2 μM TPEN, 4 μM TPEN) was evaluated daily for 72 h. Cell viability by excluding propidium iodide (PI)‐positive cells (C) MV4‐11 (*n* = 7); (D) THP‐1 (*n* = 4) and zinc staining by using Zinpyr‐1 (E) MV4‐11 (*n* = 6); (F) THP‐1 (*n* = 4) after 72 h are indicated. Zinpyr‐1 staining is indicated as mean fluorescence intensity (MFI). mRNA expression of *ZIP10* after 72 h in (G) MV4‐11 (*n* = 5) and (H) THP‐1 (*n* = 4). MTT assay over 4 h with (I) MV4‐11 cells (*n* = 4) or (J) THP‐1 cells (*n* = 7) after 72 h of cell growth after the respective cell treatment. mRNA expression of *BAX* (K) MV4‐11 (*n* = 8); (L) THP‐1 (*n* = 3) after 72 h. Two‐way ANOVA—Dunnett's multiple comparison (A + B) OR one‐way ANOVA—Dunnett's multiple comparison (C–L) were used to calculate statistical significance. Data are displayed as mean + standard deviation (SD). Significance is indicated by *p < 0.05, **p < 0.01, ***p < 0.001 and ****p < 0.0001.

To further examine the importance of zinc homeostasis in the context of AML, we used cell lines originating from an (biphenotypic) acute monoblastic/monocytic leukaemia (MV4‐11) and an acute monocytic leukaemia (THP‐1). The cells were supplemented with zinc sulphate or treated with the cell‐permeable zinc‐depleting molecule N,N,N′,N′‐tetrakis(2‐pyridinylmethyl)‐1,2‐ethanediamine (TPEN) for 72 h. The cell count was examined daily for MV4‐11 (Figure 3A) and THP‐1 (Figure 3B). After 72 h of incubation, MV4‐11 cells showed significantly decreased cell counts after incubation with 100 μM zinc sulphate (*p* = 0.0113) or 4 μM TPEN (*p* = 0.0399) (Figure S4A). THP‐1 cells exhibited a significantly decreased cell count after 72 h of incubation with 4 μM TPEN (*p* = 0.0033) (Figure S4B). Cell viability was significantly reduced in both MV4‐11 cells (Figure 3C, *n* = 7, *p* = 0.0472) and THP‐1 cells (Figure 3D, *n* = 4, *p* = 0.0005) after 72 h incubation with 4 μM TPEN. We detected significantly decreased intracellular zinc levels after incubation with 4 μM TPEN in MV4‐11 cells (Figure 3E, *n* = 6, *p* < 0.0001) and THP‐1 cells (Figure 3F, *n* = 4, *p* = 0.0134) proving efficient zinc chelation.

After 72 h supplementation with 50 μM (*p* = 0.0401) or 100 μM zinc (*p* = 0.0163), we found decreased *ZIP10* expression in MV4‐11 cells (*n* = 5) (Figure 3G). On the other hand, zinc depletion with 4 μM TPEN caused increased mRNA expression of *ZIP10* (*p* < 0.0001) (Figure 3G). Comparable results were found in THP‐1 cells (*n* = 4) treated with 100 μM zinc (*p* = 0.0382) or 4 μM TPEN (*p* = 0.5060) (Figure 3H). By applying an MTT assay, we found reduced metabolic activity in MV4‐11 cells (*n* = 4) after incubation with 100 μM zinc sulphate (*p* = 0.0065) or 4 μM TPEN (*p* = 0.0029) (Figure 3I) and in THP‐1 cells after incubation for 72 h with 4 μM TPEN (*n* = 7) compared to the control (Figure 3J, *p* = 0.0002). Concordant with the reduction in cell count and viability, we analysed the expression of the proapoptotic factors *BAX* and *BAK*. *BAX* expression was significantly increased in MV4‐11 cells after incubation with 4 μM TPEN (Figure 3K; *p* = 0.0020; *n* = 8). Increased expression of *BAX* in THP‐1 cells (Figure 3L; *p* = 0.9997; *n* = 3), *BAK* in MV4‐11 (Figure S4C; *p* = 0.3716; *n* = 6) and *BAK* in THP‐1 (Figure S4D; *p* = 0.3072; *n* = 3) after 4 μM TPEN did not reach statistical significance. Thus, zinc depletion caused apoptosis and a compensatory upregulation of *ZIP10* in AML cell lines.

### Inhibitory effects of zinc‐deficient medium on AML cell lines

**FIGURE 4:**
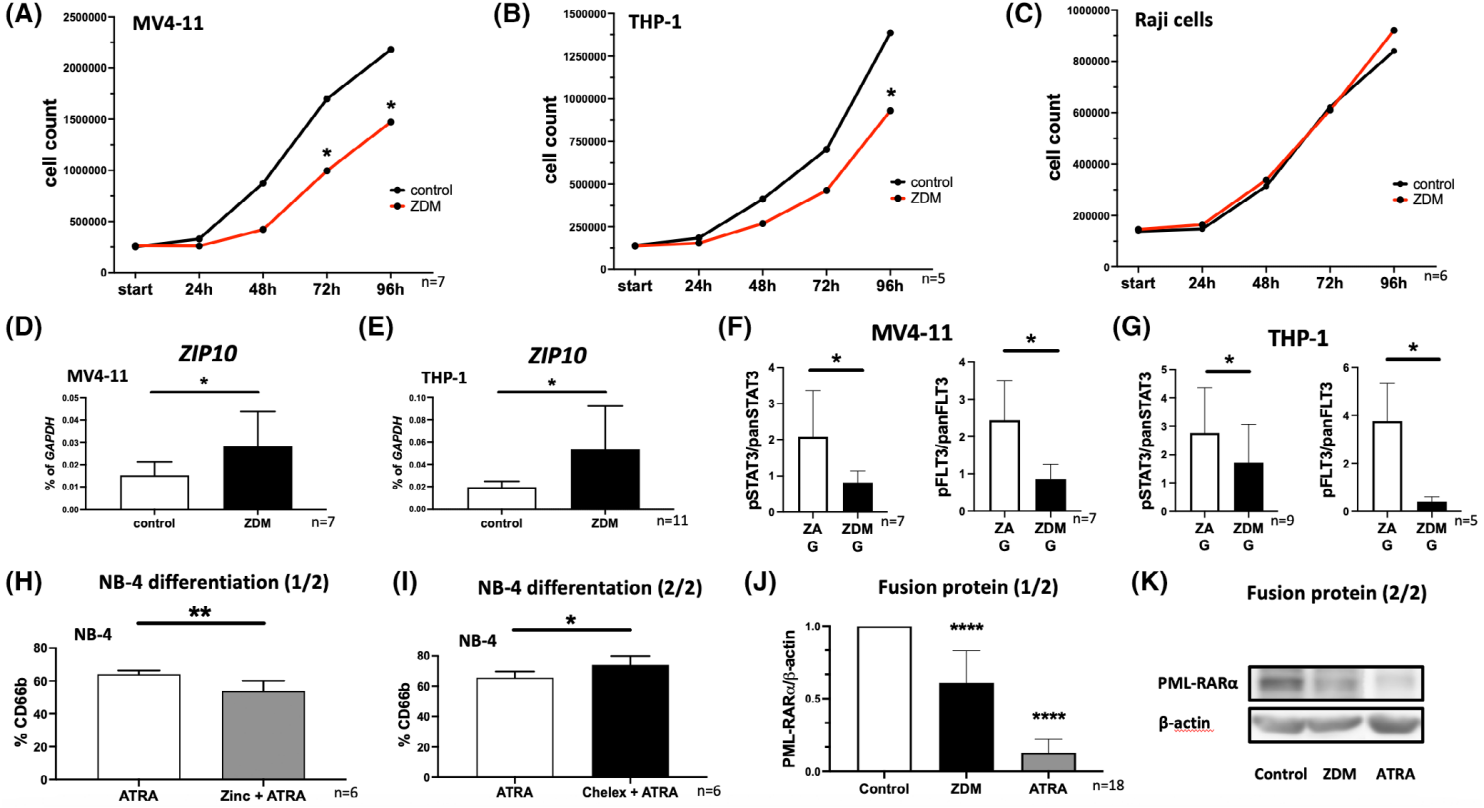
Inhibitory effects of zinc‐deficient medium on AML cell lines. Cell growth of (A) MV4‐11 (*n* = 7), (B) THP‐1 (*n* = 5) and (C) Raji cells (*n* = 6) in zinc‐adequate (ZA) or zinc‐deficient medium (ZDM) for 92 h. mRNA expression of *ZIP10* in (D) MV4‐11 (*n* = 7) and (E) THP‐1 (*n* = 11) after 96 h in ZDM. (F + G) Western blots targeting pSTAT3/panSTAT3 and pFLT3/panFLT3 after stimulation of (F) MV4‐11 (STAT3: *n* = 7, *p* = 0.0395; FLT3: *n* = 7, *p* = 0.0103) or (G) THP‐1 cells (STAT3: *n* = 9, *p* = 0.0162; FLT3: *n* = 5, *p* = 0.0122) with 2000 U/mL G‐CSF for 45 min (‘G’) after cultivation in RPMI 1640 or ZDM. (H–K) CD66b expression of NB‐4 cells were detected after 5 days of cell differentiation by using 1 μM ATRA (H) with or without 50 μM zinc sulphate (*p* = 0.0092) or (I) ZDM (*p* = 0.0200). (J) Presence of PML‐RARα/β‐actin normalized to untreated NB‐4 cells after 72 h incubation in RPMI 1640 with 1 μM ATRA or ZDM (*n* = 18, ATRA: *p* < 0.0001, ZDM: *p* < 0.0001). (K) Representative western blot after 72 h of incubation with the indicated supplements. (A–C) Two‐way ANOVA—Dunnett's multiple comparison and (D–K) Student's *t*‐test were used to calculate statistical significance. Data are displayed as mean + standard deviation (SD). Significance is indicated by *p < 0.05, **p < 0.01, ***p < 0.001 and ***p < 0.0001.

Since TPEN induces strong and rather non‐physiological zinc depletion by chelating extracellular, intracellular and protein‐bound zinc, we further examined the effect of mild zinc starvation induced via zinc‐deficient medium (ZDM). We cultured MV4‐11 cells (Figure 4A; *n* = 7), THP‐1 cells (Figure 4B; *n* = 5) and Raji cells (Figure 4C; *n* = 6), a B‐cell line, for 96 h in ZDM, and observed significantly decreased cell growth by zinc starvation of MV4‐11 cells (Figure 4A; *n* = 7) after 72 h (*p* = 0.0200) and 96 h (*p* = 0.0193) as well as THP‐1 cells (Figure 4B; *n* = 5) after 96 h (*p* = 0.0278). However, Raji cells did not respond to the incubation in ZDM (Figure 4C; *n* = 6). Raji cells were used to compare myeloid cells with a lymphoid cell line. Cultivation in ZDM did not affect the cell viability of any cell line (data not shown). After 96 h of cultivation in ZDM, we found significantly elevated mRNA levels of the zinc influx transporter *ZIP10* in MV4‐11 (Figure 4D; *p* = 0.0438; *n* = 7) and THP‐1 cells (Figure 4E; *p* = 0.0207; *n* = 11) compared to cells cultured in zinc‐adequate medium.

### Zinc is an important factor for cell signalling and the block of myeloid cell differentiation

To further examine the impact of zinc deficiency on AML cell signalling, we cultured MV4‐11 and THP‐1 in ZDM and, subsequently, stimulated both with 2000 U/mL G‐CSF for 45 min. G‐CSF was chosen to accentuate myeloid signalling as one of the most important haematopoietic pathways. We found significantly decreased G‐CSF‐induced phosphorylation of STAT3 and FLT3 in zinc‐deficient MV4‐11 (Figure 4F, STAT3: *n* = 7, *p* = 0.0395, FLT3: *n* = 7, *p* = 0.0103) and THP‐1 cells (Figure 4G, STAT3: *n* = 9, *p* = 0.0162, FLT3: *n* = 5, *p* = 0.0122). Even zinc deficiency alone resulted in decreased phosphorylation of STAT3 and FLT3 in MV4‐11 and THP‐1 (data not shown). Interestingly, STAT5 phosphorylation was significantly decreased in MV4‐11 cells, which harbour the *FLT3* ^*ITD*^ mutation (Figure S4E; *n* = 3; *p* = 0.0033), but remained unchanged in THP‐1 cells (Figure S4F; *n* = 3; *p* = 0.6957). Furthermore, we found significantly increased phosphatase activity in THP‐1 cells (Figure S4G, *n* = 12, *p* = 0.0023) explaining decreased phosphorylation under zinc‐deficient conditions. Telomerase activity was not impaired by zinc deficiency (Figure S4H).

Apart from cell proliferation, we also focused on the zinc‐dependent block of cell differentiation. We used NB‐4 cells that have the PML‐RARα translocation as its essential malignant aberration.^20^ The PML‐RARα translocation is characteristic of acute promyelocytic leukaemia (AML FAB M3) and treatment with all‐trans retinoic acid (ATRA) can be used to abolish the differentiation block. NB‐4 cells were pre‐incubated for 2 days in RPMI 1640 (control) or ZDM. After 3 and 5 days, differentiation was evaluated by measuring CD66b surface staining. We found decreased CD66b staining of NB‐4 cells under zinc‐rich conditions. Increased CD66b staining was significant after 3 (Figure S4I, *n* = 9, *p* = 0.0295) and 5 days (Figure 4H, *n* = 6, *p* = 0.0092). Zinc deficiency increased cell differentiation after 3 (Figure S4J, *n* = 8, *p* = 0.0390) and 5 days (Figure 4I, *n* = 6, *p* = 0.0200). The efficiency of zinc supplementation (Figure S5A) and zinc deprivation (Figure S5B,C) were proven by intracellular zinc staining. During the differentiation process itself, we saw decreasing intracellular zinc levels (Figure S5D). Furthermore, we analysed the presence of the PML‐RARα fusion protein after zinc deficiency. After 72 h in ZDM, we found decreased (relative) levels of PML‐RARα (normalized to β‐actin) (*p* = <0.0001; *n* = 18), which showed a comparable but less effective effect to the treatment of NB‐4 cells with 1 μM ATRA (39% vs. 87% reduction, *n* = 18, *p* = 0.0001) (Figure 4J). Increased PML‐RARα degradation by zinc deficiency is also presented by a representative western blot (Figure 4K).

### Impact of a zinc‐deficient environment on primary AML blasts

**FIGURE 5:**
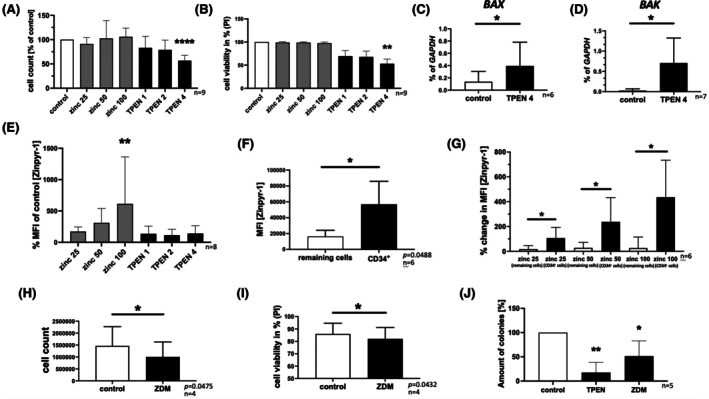
Impact of a zinc‐deficient environment on primary AML blasts. Primary peripheral blood cells or bone marrow cells from patients with first diagnosis of acute myeloid leukaemia (AML) were used. Blast concentration differed from 20% to 87%. (A) Cell concentration in relation to the control condition after incubation for 72 h with zinc sulphate (25 μM, 50 μM, 100 μM) or N,N,N′,N′‐tetrakis(2‐pyridinylmethyl)‐1,2‐ethanediamine (TPEN) (1 μM, 2 μM, 4 μM) to cause zinc depletion (*n* = 9). (B) Cell viability was measured using propidium iodide (PI) (*n* = 9). mRNA expression of (C) *BAX* (*n* = 6) and (D) *BAK* (*n* = 7) after 72 h. (E) Measurement of the free intracellular zinc concentration of primary patient materials by using Zinpyr‐1 staining after 72 h of incubation with zinc sulphate (25 μM, 50 μM, 100 μM) or TPEN (1 μM, 2 μM, 4 μM) (*n* = 8). (F) Free intracellular zinc in CD34^+^ cells compared to CD34‐negative cells (remaining cells) after 72 h of incubation by comparing the mean fluorescence intensity (MFI) of the zinc dye Zinpyr‐1 (*n* = 6). (G) Influx of zinc into CD34^+^ cells (black coloured) compared to CD34‐negative cells (remaining cells) (white coloured) (*n* = 6) by using % MFI after incubation for 72 h with zinc sulphate (25 μM, 50 μM, 100 μM). (H) Cell concentration and (I) cell viability by using propidium iodide (PI) after incubation of primary AML blasts in RPMI 1640 or zinc‐deficient medium (ZDM) for 72 h. (J) Primary bone marrow cells from AML patients were treated with TPEN [4 μM] or cultured in ZDM for 72 h. Cell viability was evaluated by using PI. For every condition, 5000–50 000 viable cells were applied into semi‐solid medium to perform a CFU assay. After 14 days, colonies were counted and normalized to control. (A + B + E + J) One‐way ANOVA—Dunnett's multiple comparison and (C + D + F–I) Student's *t*‐test was used to calculate statistical significance. Data are displayed as mean + standard deviation (SD). Significance is indicated by *p < 0.05, **p < 0.01, ***p < 0.001 and ***p < 0.0001.

To prove that zinc deficiency also has a relevant impact on primary AML blasts, we performed similar experiments with patient material. Bone marrow mononuclear cells (BMMCs) or PBMCs from newly diagnosed AML patients that were incubated for 72 h with 4 μM TPEN showed significantly lower cell counts (Figure 5A; *n* = 9; *p* < 0.0001) and decreased cell viability (Figure 5B; *n* = 9; *p* = 0.0061). Additionally, we found significantly increased mRNA expression of *BAX* (Figure 5C; *n* = 6; *p* = 0.0446) and *BAK* (Figure 5D; *n* = 7; *p* = 0.0266). Also, significantly increased intracellular zinc was detected after zinc supplementation (100 μM zinc: *p* = 0.0081; *n* = 8) (Figure 5E). The co‐staining of PBMCs from AML patients with Zinpyr‐1 and LysoTracker (or MitoTracker) demonstrated that Zinpyr‐1 co‐localizes within the lysosomal compartment (Figure S5E), but not the mitochondrial (Figure S5F). Cells with blast morphology showed positive surface staining for CD34 and ZIP10 as revealed using microscopy (Figure S6A) and flow cytometry (Figure S6B). By looking at the amount of free intracellular zinc in CD34^+^ cells, we found that CD34^+^ cells had significantly higher mean fluorescence intensity (MFI) in Zinpyr‐1 fluorescence compared to CD34‐negative cells (‘remaining cells’) (Figure 5F, *n* = 6, *p* = 0.0488). This is in line with our previous measurements of (whole) cellular zinc amount (Figure 1C,D). Strikingly, we found significantly higher zinc uptake after 72 h of culture in zinc‐supplemented media in CD34^+^ cells compared to CD34^−^ cells for all tested zinc concentrations (*n* = 6) (Figure 5G).

The increase of ZIP10 and zinc uptake of malignant CD34^+^ cells may be a sign of special dependency of these cells to zinc nutrition. Therefore, we incubated primary patient material for 72 h in ZDM (a milder model of zinc deficiency) and found decreased cell counts (Figure 5H; *p* = 0.0475) as well as decreased cell viability (Figure 5I; *p* = 0.0432; *t*‐test). Specifically, CD34^+^ cells treated with 4 μM TPEN (Figure S6C; *n* = 4; *p* = 0.0130) or ZDM (Figure S6D; *n* = 3; *p* = 0.0145) showed increased cytotoxicity compared to CD34^−^ cells. Furthermore, we observed a decreased percentage of CD34^+^ cells among all cells after 72 h in ZDM (Figure S6E, *n* = 4; *p* = 0.0492), whereas no significant change was detected in the percentage of CD3^+^ cells (Figure S6F; *n* = 4; *p* = 0.8974). If CD34^+^ blasts are particularly targeted by zinc depletion, clonogenic growth should be decreased upon treatment. Therefore, we performed CFU assays using equal cell numbers after 72 h of TPEN treatment or ZDM cell culture. After 14 days, colony growth was significantly decreased in zinc‐deficient conditions (Figure 5J; 4 μM TPEN: *n* = 5, *p* = 0.0016; ZDM: *n* = 5, *p* = 0.0443).

### Treatment of AML cell lines with a ZIP10 antibody

**FIGURE 6:**
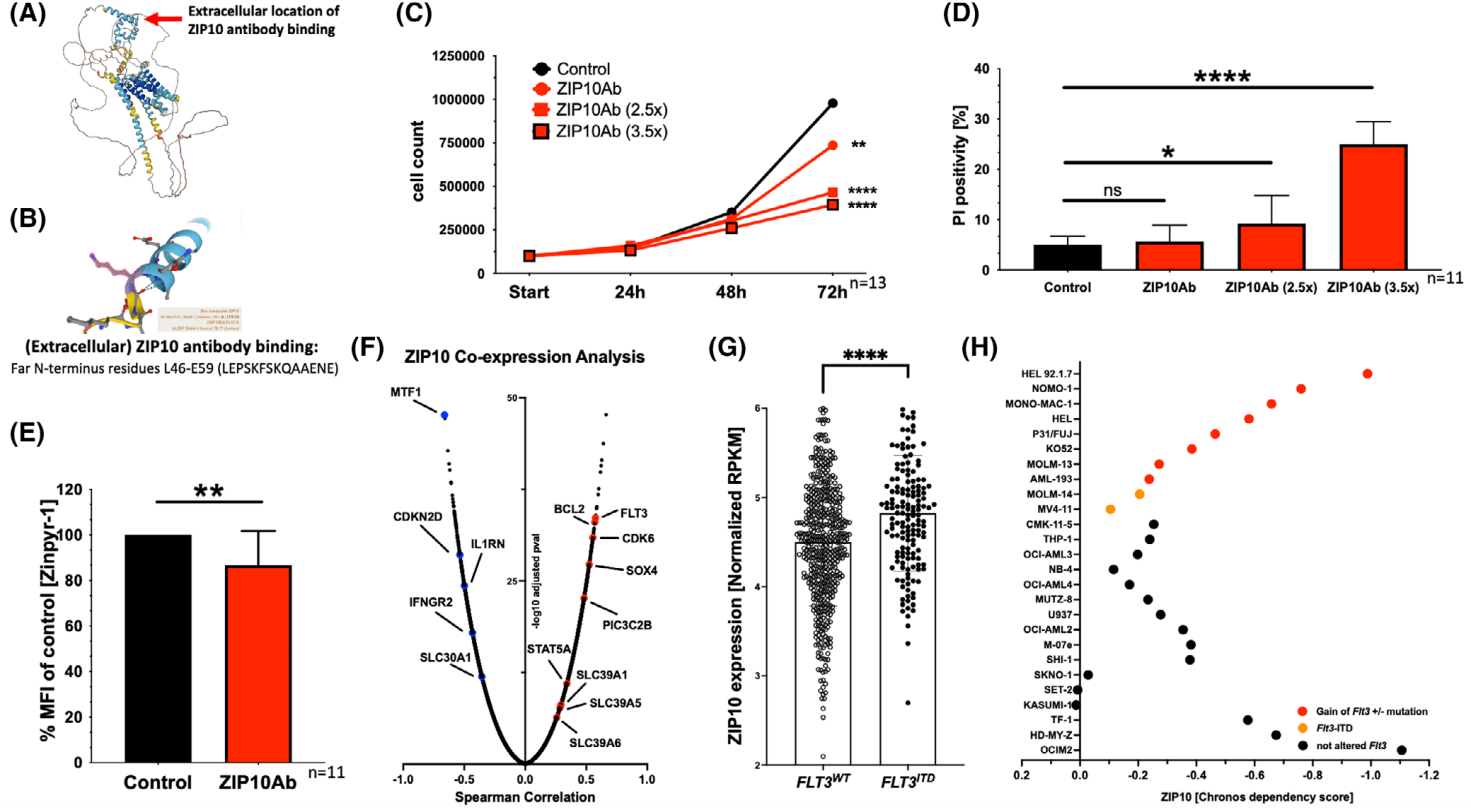
Evaluation of the efficacy of a ZIP10 antibody as a therapeutic molecule in acute myeloid leukaemia (AML). Dependence of AML on the ZIP10 protein. (A) Predicted protein structure of ZIP10 by using AlphaFold^22^, ^23^ and the (B) binding region of the experimentally used ZIP10 antibody (ZIP10Ab) that is essential for zinc uptake required to trigger mitosis. (C) Cell count after treatment of MV4‐11 cells with 8 μg/mL (1×), 20 μg/mL (2.5×) and 28 μg/mL (3.5×) purified ZIP10Ab. After 72 h, cell count (*n* = 13) was significantly different compared to controls that were treated with the highest concentration of elution buffer (ZIP10Ab: *p* = 0.0092; ZIP10Ab (2.5×): *p* < 0.0001 and ZIP10Ab (3.5×): *p* < 0.0001). (D) PI‐positivity (*n* = 11) after 72 h treatment with ZIP10Ab (*p* = 0.9587), ZIP10Ab (2.5×) (*p* = 0.0477) and ZIP10Ab (3.5×) (*p* < 0.0001). (E) Zinpyr‐1 staining in MV4‐11 cells (*n* = 11) after treatment with the purified ZIP10Ab for 72 h (*p* = 0.0081). (F) Co‐expression analysis of *ZIP10* in AML patients.^18^, ^19^ (G) *ZIP10* expression in AML patients with *FLT3* ^*ITD*^ compared to patients with wildtype *FLT3* included in the BeatAML2.0 study^18^, ^19^ (*FLT3* ^*wt*^: *n* = 511; *FLT3* ^*ITD*^: *n* = 158; *p* < 0.0001). (H) Dependency of various AML cell lines on the ZIP10 (surface) protein (a negative Chronos dependency score implies a higher probability that the *ZIP10* gene is indispensable for the respective cell line). AML cell lines with a known copy number (absolute) in the *Flt3* gene were selected from the depmap portal (red‐marked: ≥3 copies).^24^, ^25^, ^26^, ^27^ (C) Two‐way ANOVA—Dunnett's multiple comparison. (D) One‐way ANOVA—Dunnett's multiple comparison, and (E + G) Student's *t*‐test was used to calculate statistical significance. Data are displayed as mean + standard deviation (SD). Significance is indicated by *p < 0.05, **p < 0.01, ***p < 0.001 and ****p < 0.0001.

Since *ZIP10* was one of the most upregulated zinc transporters in AML (Figure 2C) and it has been demonstrated to be essential for mitosis,^21^ we further examined whether blocking zinc transport via ZIP10 impacts AML cell viability. Unfortunately, current market‐available antibodies that target the opening region of the ZIP10 transport protein contain the preservative sodium azide, which is toxic for cells and was therefore not applicable for our in vitro experiments.^22^, ^23^ We used a ZIP10 antibody (ZIP10Ab) that binds to the (extracellular) opening region of the ZIP10 protein (Figure 6A,B) thereby impairing zinc uptake as previously described.^21^ Therefore, we treated MV4‐11 cells with 8 μg/mL, 20 μg/mL (2.5×) and 28 μg/mL (3.5×) of a purified ZIP10Ab. After 72 h, cell growth was gradually decreased depending on the administered antibody concentration ZIP10Ab (*p* = 0.0092), ZIP10Ab (2.5×) (*p* < 0.0001) and ZIP10Ab (3.5×) (*p* < 0.0001) (Figure 6C). Moreover, treatment with ZIP10Ab (2.5×) (*p* = 0.0477) and ZIP10Ab (3.5×) (*p* < 0.0001) resulted in significantly increased number of dead AML cells (Figure 6D). Mechanistically, we found decreased intracellular zinc staining after 72 h of treatment (Figure 6E; *p* = 0.0081). Treatment with the same amount of elution buffer showed no impact on cell count after 72 h (Figure S7A). Successful surface binding of the ZIP10 antibody was proven after 24 h and 72 h (Figure S7B,D).

### Dependency of AML on the ZIP10 protein

Co‐expression analyses of *ZIP10* in AML patients showed positive correlations with genes that are associated with proliferation and apoptosis resistance, such as *FLT3*, *BCL2*, *CDK6* and *SOX4* (Figure 6F). Interestingly, patients with pathogenic changes in the *FLT3* gene such as *FLT3* ^*ITD*^ showed significantly higher *ZIP10* expression compared to other AML patients (Figure 6G). Since *FLT3* ^*ITD*^ is associated with increased proliferation and survival in AML cells,^28^ we assessed the dependency of different AML cell lines in regard to the ZIP10 protein. In an extensive knock‐out CRISPR screen dataset,^24^, ^25^, ^26^, ^27^ most AML cell lines showed a strong dependency on the ZIP10 protein indicated by a negative Chronos dependency score (Figure 6H). Cell lines that harbour a proven aberration in *FLT3* (red + orange) showed, by tendency, a stronger dependency on ZIP10 (Figure 6H, *p* = 0.1863).

## DISCUSSION

Zinc is a micronutrient which is crucial for diverse cellular processes and has been discussed to be important for the survival of different cancer cell types, such as breast cancer.^21^, ^29^ In this study, we demonstrate that both total cellular zinc as well as free intracellular zinc were significantly elevated in AML cells compared to controls, suggesting altered zinc metabolism in AML blasts (see also Appendix S2). We focused on ZIP10 as it showed low expression in almost all control samples and significant overexpression in AML cases (Figure S3 + Figures [Link], [Link], [Link]).

Zinc deprivation decreased proliferation and viability of AML cell lines and primary blasts. Interestingly, we found no significant impairment of cell growth under zinc‐deficient conditions in a malignant B‐cell line. Mechanistically, we saw that severe intracellular zinc depletion by TPEN treatment caused apoptosis in MV4‐11 and THP‐1. To counteract zinc deficiency, AML cells upregulated *ZIP10*. We also identified another mechanism involving reduced STAT3, FLT3 and STAT5 phosphorylation under zinc‐deficient conditions. Moreover, zinc deprivation has been shown to promote cell differentiation into monocytes,^30^, ^31^ a process that is of particular relevance given that several subtypes of AML exhibit monocyte‐like morphology.^32^ Consistent with these findings, we observed similar effects when applying zinc deprivation to the promyelocytic leukaemia cell line NB‐4.

Targeting the ZIP10 influx transporter on the cell surface effectively inhibited AML cell growth by disrupting intracellular zinc uptake, or at the very least, by preventing zinc accumulation near the transporter, thereby impairing mitosis.^21^ As previously suggested,^21^, ^33^ ZIP10 may be primarily and predominantly expressed on the surface of proliferating cells. Given that ZIP10 and ZIP6 can form a heterodimer to synergistically promote zinc‐mediated mitosis, it is especially noteworthy that both are overexpressed in AML patients.^21^ Consistent with our study, it was shown that ZIP10 is an essential (transport) protein for haematopoiesis in zebrafish and loss of ZIP10 resulted in zinc deficiency‐induced apoptosis of fetal HSCs.^34^

In summary, we show that AML blasts have higher zinc levels with concurrent upregulation of transporters important for zinc uptake. Furthermore, targeting the upregulated ZIP10 transporter decreased AML cell proliferation. This prompts future studies to explore whether monitoring zinc levels and modulating zinc homeostasis could provide a mechanistic approach to optimize AML therapy and diminish the risk of relapse after allo‐SCT. Our study primarily highlights ex vivo effects. Hence, future in vivo studies will be valuable in further exploring and confirming these observations.

## AUTHOR CONTRIBUTIONS

BR, NC, RG, MV, NT‐T, JB and IW conducted experiments. The project was planned by BR, THB, LR and IW. Statistical analyses were done by BR, NC, RG, MV, NTT, JB and IW. Publicly available data were analysed by BR, ACH, KAR and IW. Clinical samples were provided by BR, MGB, DC and JW. Clinical input was provided by BR, MGB, DC, JW, EJ, SK, FB, MS and THB. Scientific input was provided by NC, KAR, KMT, EJ, SK, FB, MS, THB, LR and IW. The manuscript was drafted by BR and edited by all (co‐)authors.

## FUNDING INFORMATION

Benjamin Rolles received a Mildred‐Scheel scholarship from the German Cancer Aid (‘Deutsche Krebshilfe’) (No. 70114570).

## CONFLICT OF INTEREST STATEMENT

The authors declare no conflict of interest. KMT is the named inventor on a patent describing the use of ZIP6 and ZIP10 antibodies to inhibit mitosis and stands to gain from their development.

## PUBLIC AVAILABLE DATASETS

Additional AML data were acquired from the Leukemia Mile study (BloodSpot) (https://servers.binf.ku.dk/bloodspot/)^15^, ^16^, ^17^ and the BeatAML2.0 study (Vizome) (http://www.vizome.org/aml2/).^18^, ^19^ Public available data on protein structure were used from AlphaFold (https://alphafold.ebi.ac.uk).^22^, ^23^ Data from CRISPR knock‐out screens were used from the DepMap Public 23Q4 dataset (Broad Institute) (https://depmap.org).^24^, ^25^, ^26^, ^27^

## Supporting information


Figure S1.

Figure S2.

Figure S3.

Figure S4.

Figure S5.

Figure S6.

Figure S7.

Figure S8.

Figure S9.

Figure S10.

Table S1.

Table S2.

Appendix S1.

Appendix S2.

## Data Availability

The data will be provided on reasonable request.
