# Supplementary figures and images for: ZIP10 as a potential therapeutic target in acute myeloid leukaemia
Source: Br J Haematol. 2025 Jun 30;207(3):767–79. doi: 10.1111/bjh.20229 (PMC12436223; doi:10.1111/bjh.20229)

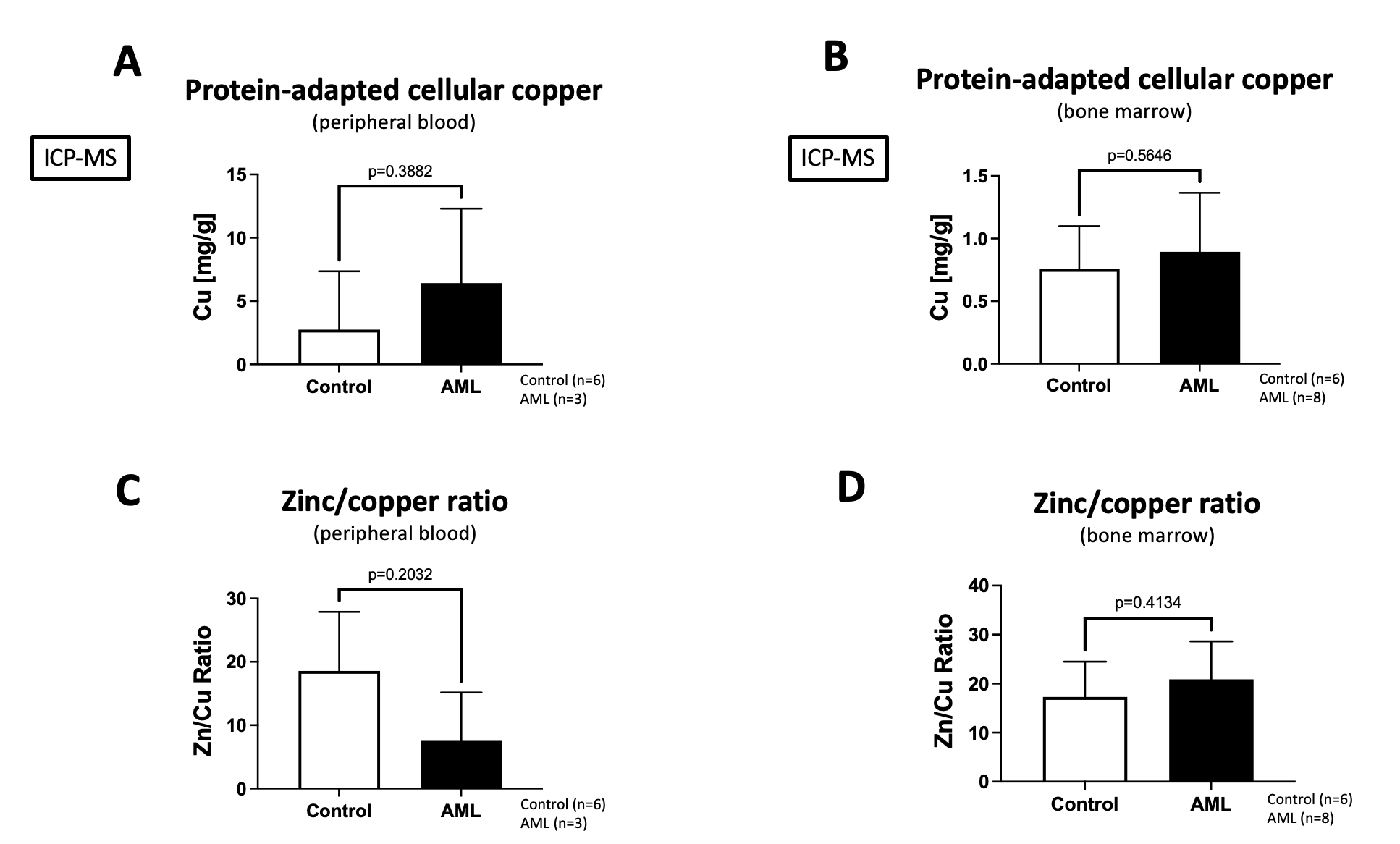

Supplement: Supplementary file 1 — Figure S1. [file BJH-207-767-s010.png]

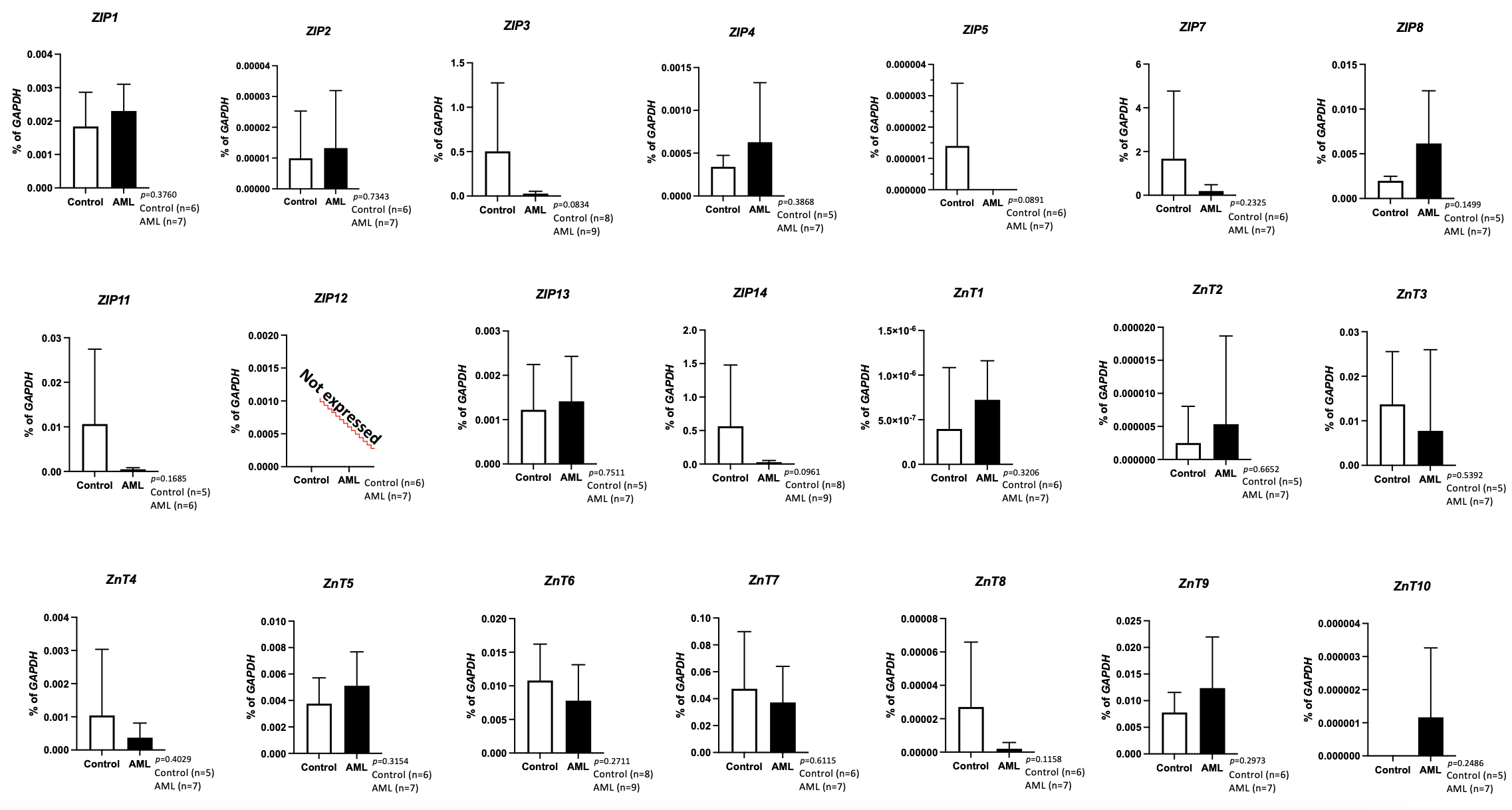

Supplement: Supplementary file 2 — Figure S2. [file BJH-207-767-s003.png]

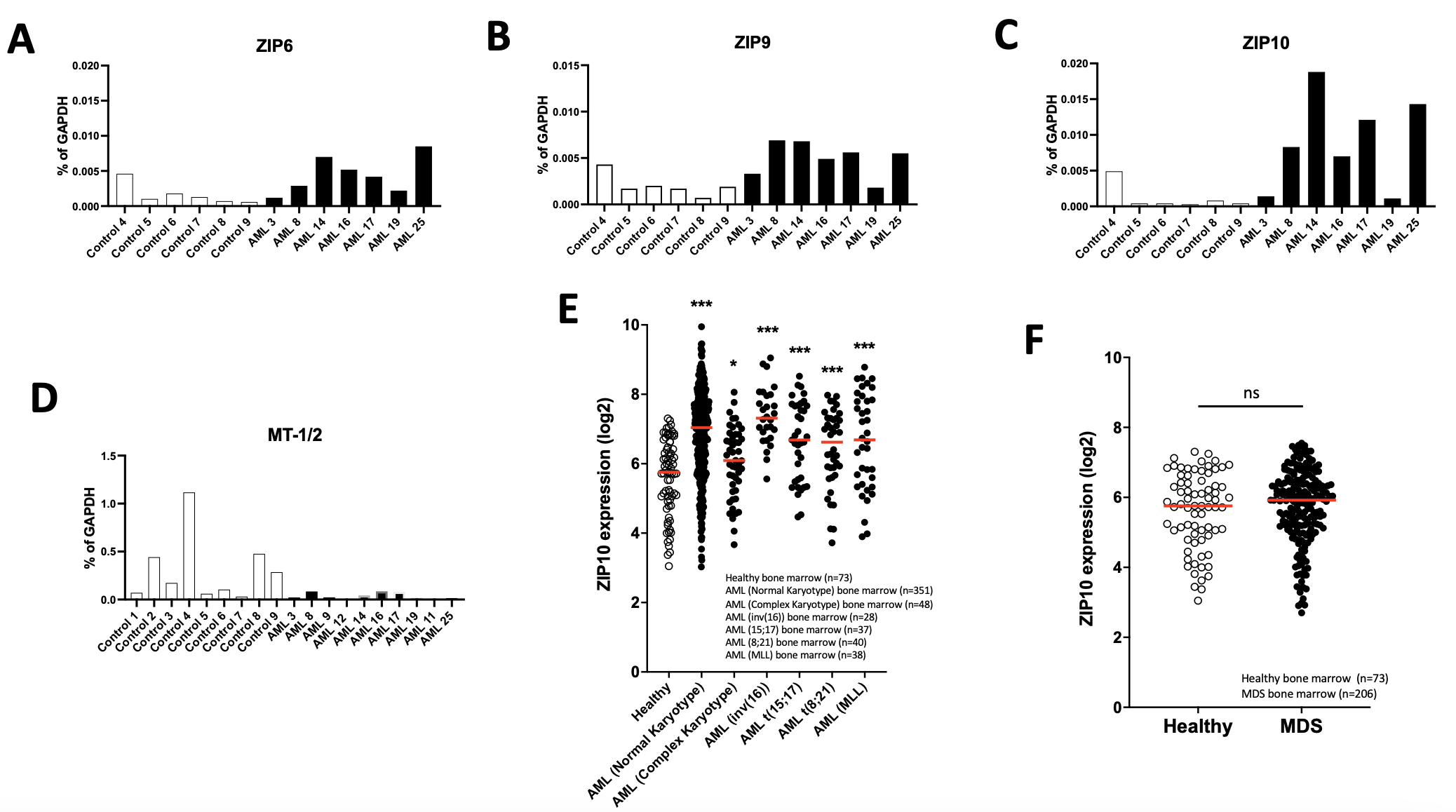

Supplement: Supplementary file 3 — Figure S3. [file BJH-207-767-s007.png]

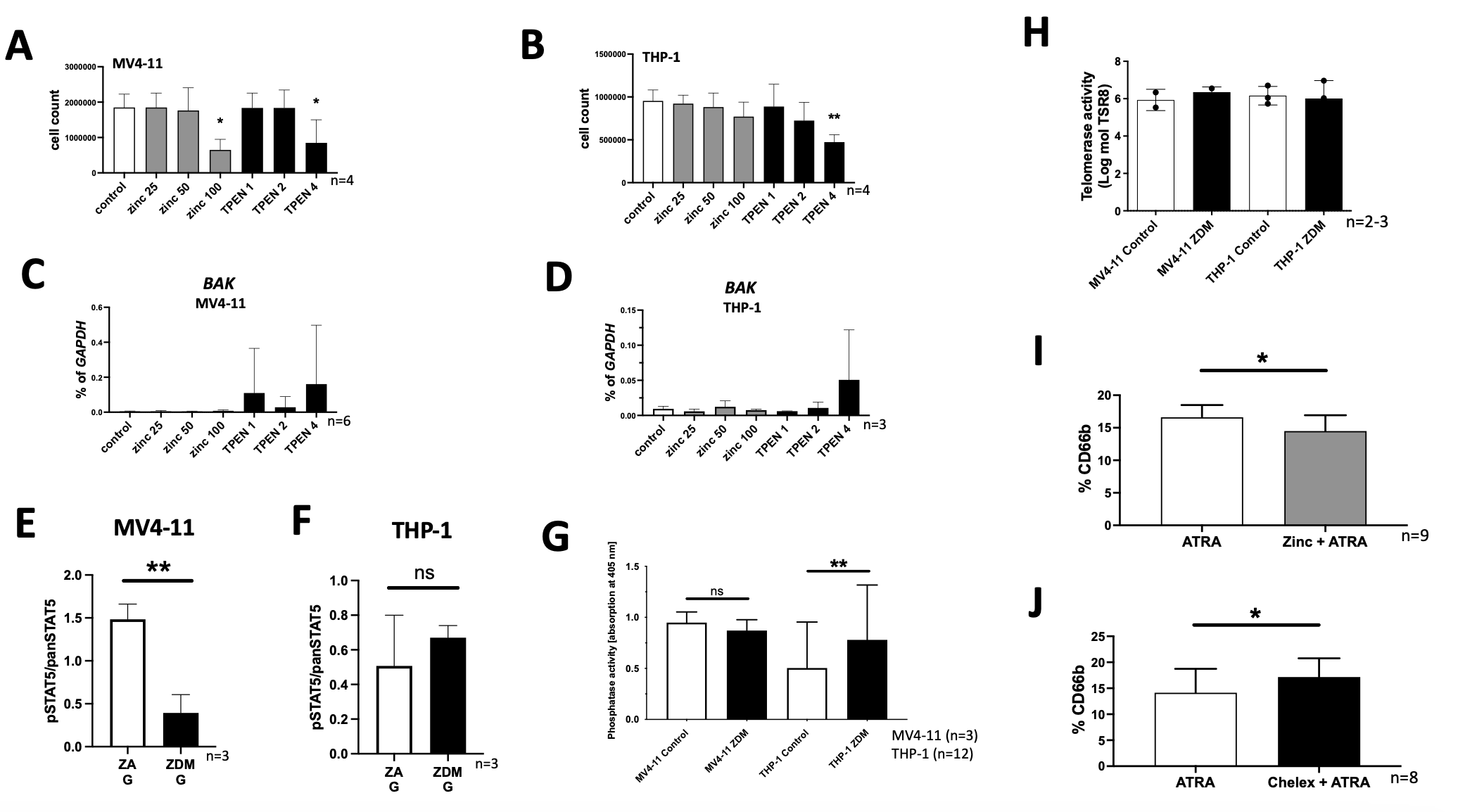

Supplement: Supplementary file 4 — Figure S4. [file BJH-207-767-s005.png]

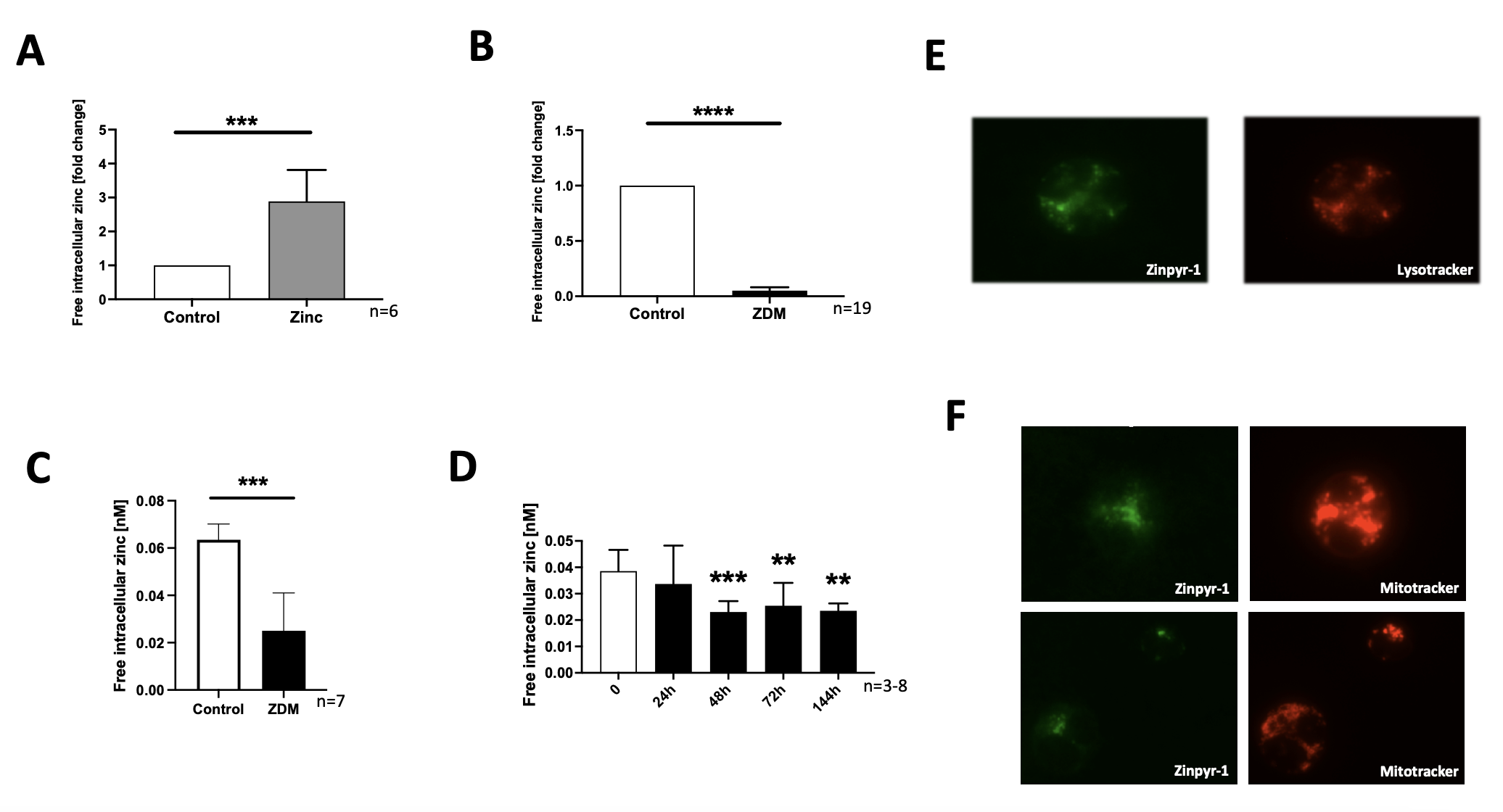

Supplement: Supplementary file 5 — Figure S5. [file BJH-207-767-s001.png]

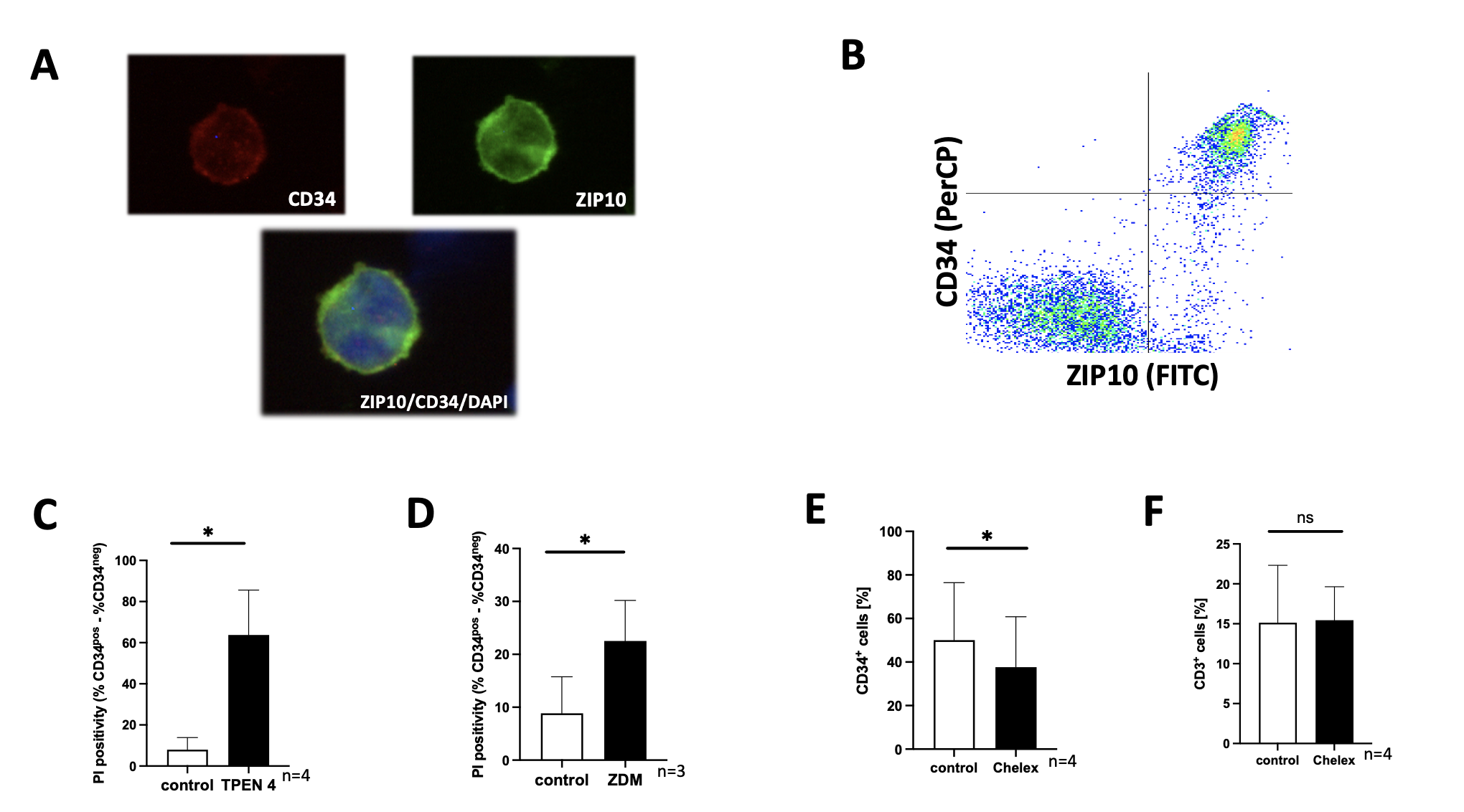

Supplement: Supplementary file 6 — Figure S6. [file BJH-207-767-s013.png]

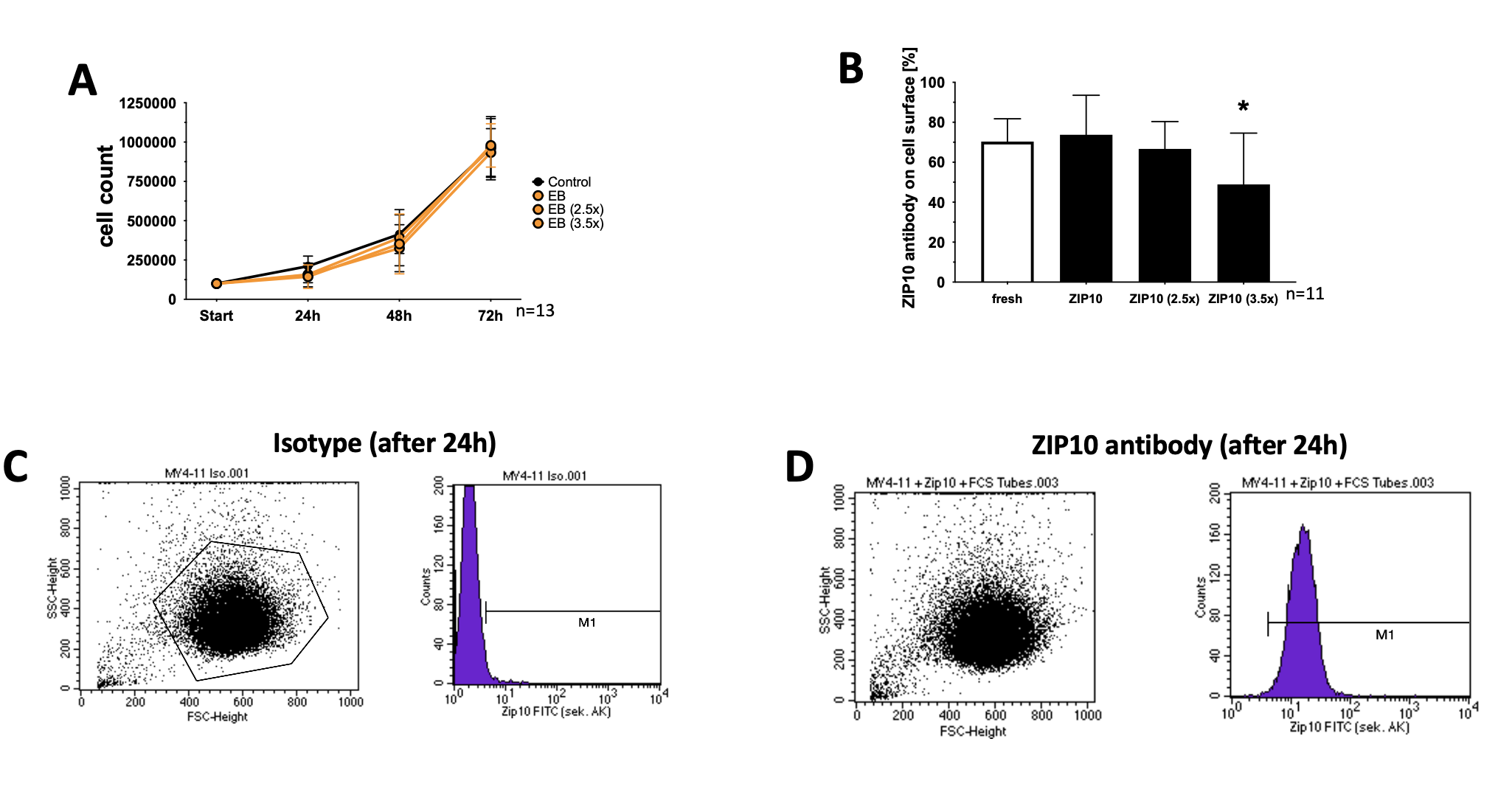

Supplement: Supplementary file 7 — Figure S7. [file BJH-207-767-s006.png]

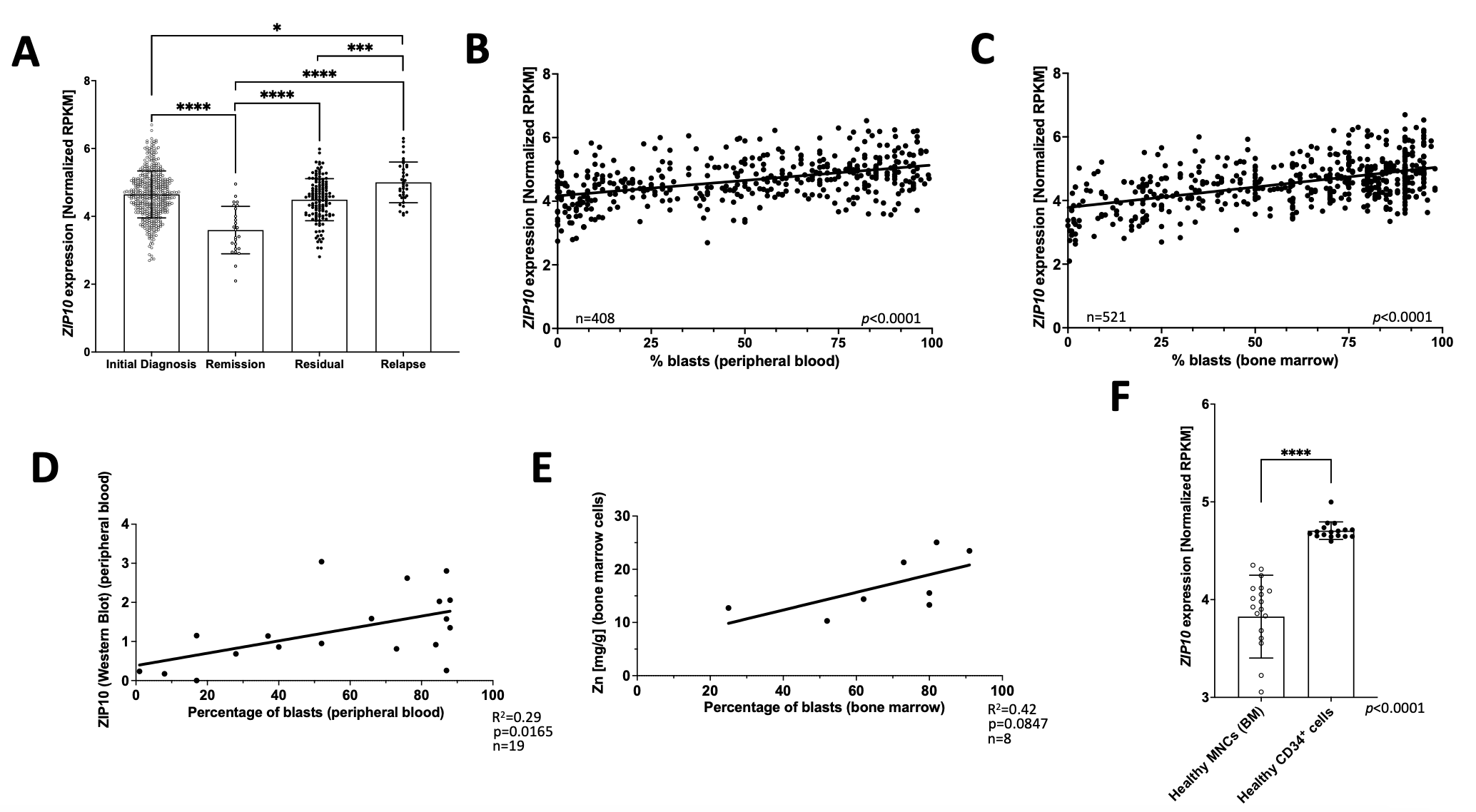

Supplement: Supplementary file 8 — Figure S8. [file BJH-207-767-s008.png]

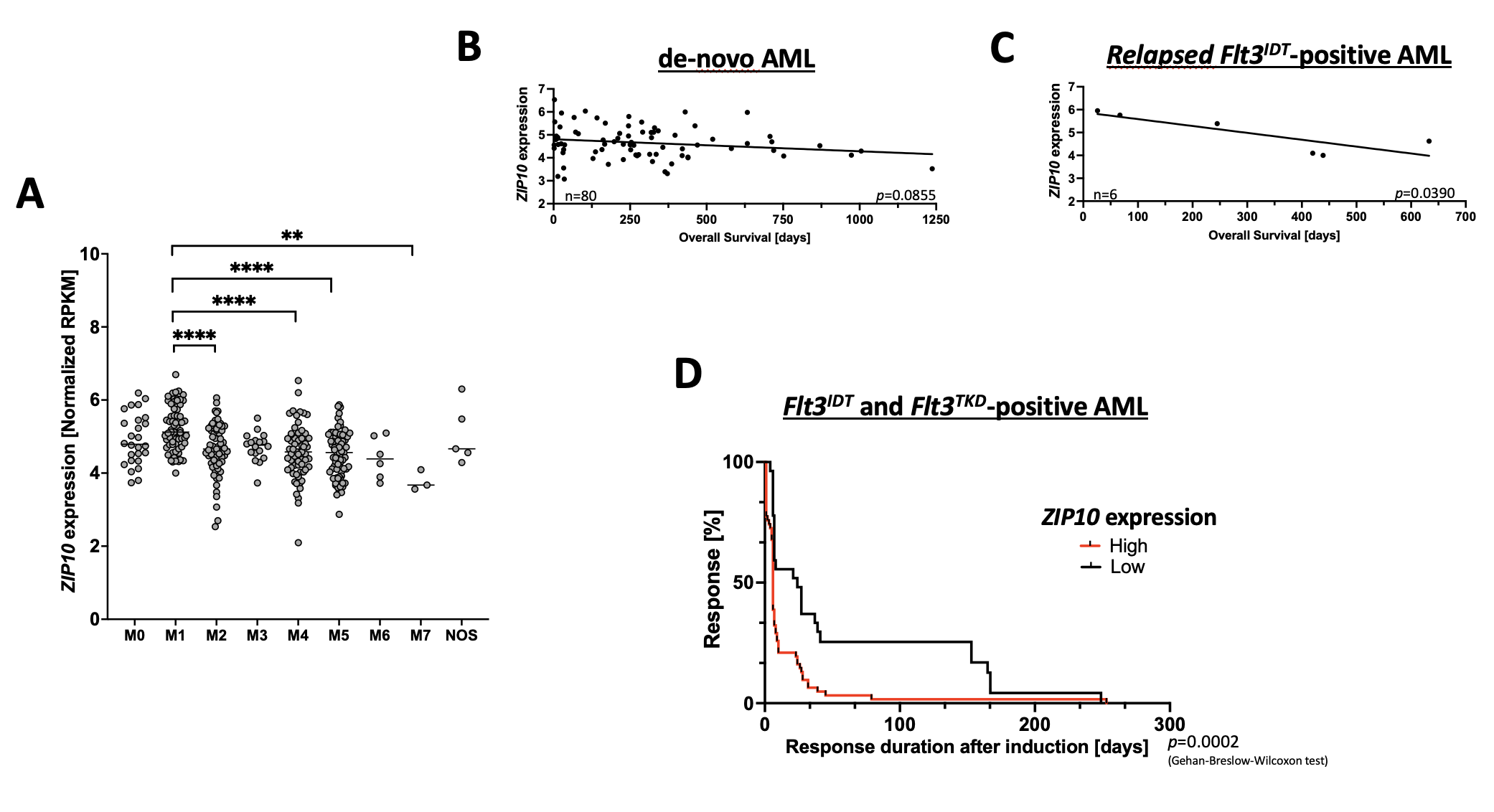

Supplement: Supplementary file 9 — Figure S9. [file BJH-207-767-s011.png]

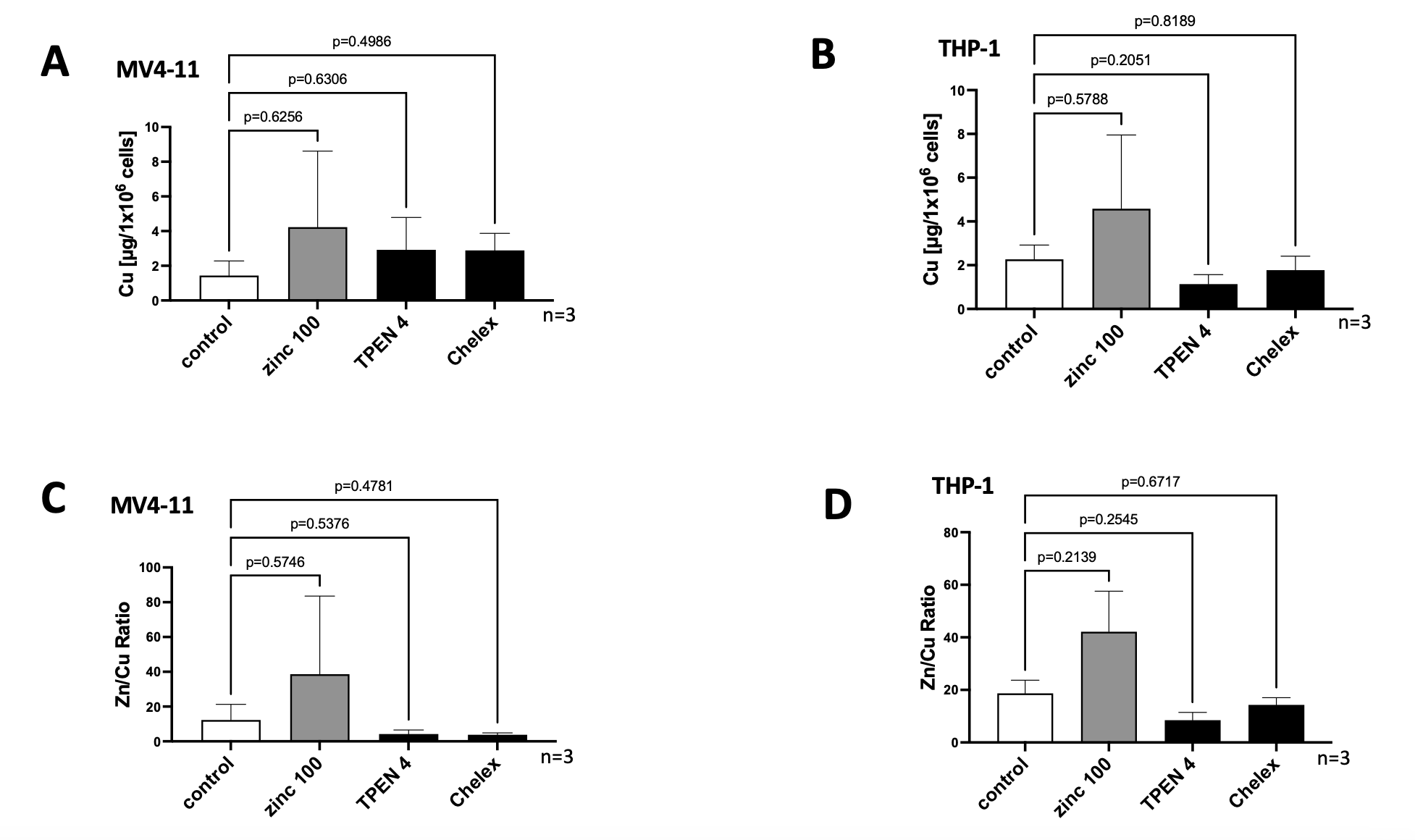

Supplement: Supplementary file 10 — Figure S10. [file BJH-207-767-s014.png]
