# Supplementary material for: ZIP10 as a potential therapeutic target in acute myeloid leukaemia
Source: Br J Haematol. 2025 Jun 30;207(3):767–79. doi: 10.1111/bjh.20229 (PMC12436223; doi:10.1111/bjh.20229)
Supplement: Supplementary file 11 — Table S1. [file BJH-207-767-s012.docx]

| **Sample No.** | **CD34 positivity** | **Figure 1**  **(bone marrow - serum)** | **Figure 1**  **(bone marrow - whole cell lysates)** | **Figure 1**  **(peripheral blood - serum)** | **Figure 1**  **(peripheral blood - whole cell lysates)** | **Figure 2-4** | **Figure 6** |
| --- | --- | --- | --- | --- | --- | --- | --- |
| AML No.1 | pos. | no | yes | no | no | no | yes |
| AML No.2 | pos. | no | yes | no | no | no | yes |
| AML No.3 | pos. | yes | yes | yes | yes | no | no |
| AML No.4 | pos. | yes | yes | yes | no | no | no |
| AML No.5 | pos. | yes | no | yes | no | no | no |
| AML No.6 | pos. | yes | no | yes | no | no | no |
| AML No.7 | pos. | no | yes | no | no | no | no |
| AML No.8 | pos. | yes | no | yes | no | no | no |
| AML No.9 | pos. | yes | yes | yes | no | no | no |
| AML No.10 | pos. | yes | no | yes | yes | no | no |
| AML No.11 | pos. | yes | no | yes | no | no | no |
| AML No.12 | pos. | yes | yes | yes | yes | no | no |
| AML No.13 | pos. | yes | yes | yes | no | no | no |
| AML No.14 | pos. | yes | no | yes | no | no | no |
| AML No.15 | pos. | no | no | no | no | yes | no |
| AML No.16 | pos. | no | no | no | no | yes | no |
| AML No.17 | pos. | no | no | no | no | yes | no |
| AML No.18 | pos. | no | no | no | no | yes | yes |
| AML No.19 | pos. | no | no | no | no | yes | no |
| AML No.20 | pos. | no | no | no | no | yes | no |
| AML No.21 | pos. | no | no | no | no | yes | yes |
| AML No.22 | pos. | no | no | no | no | yes | no |
| AML No.23 | pos. | no | no | no | no | yes | no |
| AML No.24 | pos. | no | no | no | no | yes | no |
| AML No.25 | pos. | no | no | no | no | yes | no |
| AML No.26 | pos. | no | no | no | no | yes | no |
| AML No.27 | pos. | no | no | no | no | yes | no |
| AML No.28 | pos. | no | no | no | no | yes | no |
| AML No.29 | pos. | no | no | no | no | yes | no |
| AML No.30 | pos. | no | no | no | no | yes | no |
| AML No.31 | pos. | no | no | no | no | yes | no |
| AML No.32 | pos. | no | no | no | no | yes | no |
| AML No.33 | pos. | no | no | no | no | yes | no |
| AML No.34 | pos. | no | no | no | no | yes | no |
| AML No.35 | pos. | no | no | no | no | yes | no |
| AML No.36 | pos. | no | no | no | no | yes | no |
| AML No.37 | pos. | no | no | no | no | yes | no |
| AML No.38 | pos. | no | no | no | no | yes | no |
| AML No.39 | pos. | no | no | no | no | no | yes |
| AML No.40 | pos. | no | no | no | no | no | yes |
| AML No.41 | pos. | no | no | no | no | no | yes |
| AML No.42 | pos. | no | no | no | no | no | yes |
| AML No.43 | pos. | no | no | no | no | no | yes |
