# Supplementary material for: ZIP10 as a potential therapeutic target in acute myeloid leukaemia
Source: Br J Haematol. 2025 Jun 30;207(3):767–79. doi: 10.1111/bjh.20229 (PMC12436223; doi:10.1111/bjh.20229)
Supplement: Supplementary file 12 — Table S2. [file BJH-207-767-s004.docx]

| **Gene** | **Forward Primer** | **Reverse Primer** |
| --- | --- | --- |
| *ZIP1* | GCC TGA CTA CCT GGC TGC CAT AGA | CGG CCC TGA CTG CTC CTT GTA A |
| *ZIP2* | GTG CAG AAC AGA TCA GCA AGT GA | CAA TGC CAG CGA CTC CAA A |
| *ZIP3* | GGA CTC TGA TGC ATT TTC AAC ACT | CCC TTG TTT GGC CGG TAG A |
| *ZIP4* | AGA CTG AGC CCA GAG TTG AGG CTA | TGT CGC AGA GTG CTA CGT AGA GGA |
| *ZIP5* | GAG CAG GAG CAG AAC CAT TAC CTG | CAA TGA GTG GTC CAG CAA CAG AAG |
| *ZIP6* | CAT AGC CAT GAA GAA CCA GCA ATG | GAG AAT CAA AGT GGG AGG GCT CTT |
| *ZIP7* | ACT GAA GGA GGA GCA GTG GAC AGT | AGG CCC TAA TGC CAA AGT AAC CAT |
| *ZIP8* | CCT CGG ATT GAT TTT GAC TCC ACT | AGC AGG ATT TGC ATA GCA TGT CAC |
| *ZIP9* | GCC TAA AGA ACT GGA AAG CCC ACT | GTG TTT CAC TTG CTT GGT GGT GTT |
| *ZIP10* | TAG CCG TCT TCT GTC ATG AAC TGC | TCA TAG AGG GCA ATC ACC AGC ATA |
| *ZIP11* | TCT CCT AAG CAT TTT GGT GGC CTA | TCT CTT CTT TCC ACA GGG CTC ACT |
| *ZIP12* | CAA CCA CTC AAG AAG CCT CAT CAA | AAG TAC TGC CTG GTG AAA GCC AAG |
| *ZIP13* | AAG AAG ATC GGG CTC CTG ACA AC | GAG AAC AGC ACC ATT ACC ACG ATG |
| *ZIP14* | CAT TTG GTT TCA ACC CTC TGG AAG | TTT CAG CCA GTA GCA AGC ACT CTG |
| *ZnT1* | GGC CAA TAC CAG CAA CTC CAA | TGC AGA AAA ACT CCA CGC ATG T |
| *ZnT2* | CTG CAC CTT CGT CTT CTC CAT | GAC AGC AGC AGA TCA CGA ACA G |
| *ZnT3* | CAC CCT CCG AGA CGT TCT TC | GGC AAC ATG GTA AGT GAG CGT AA |
| *ZnT4* | GGC TAT CAT CAA AAT CAC CAA CCA | CGG TGA TGA GCA TTA TAT CTC CAT T |
| *ZnT5* | AAG GAC ATC ATG ACA GTG CTC TAA CTC | CCA ACT TTA CAA CAC AAA GCC AGT AC |
| *ZnT6* | GGC AAG TTG TTA CGG GAA TTT AGA | TAA GCA GGA AGC CAG TAC ATA TCA A |
| *ZnT7* | CAC GGA CAC AGT CAT TCC CTC TT | TCT GTC TGC TGG GTC CTG TTG |
| *ZnT8* | TCC CTC TAA GCG GCT GAC AT | CAC AGT CGC CTG GAT CTG GTA |
| *ZnT9* | CAG AAA GAA GGA CAG GGA TCA CA | TTC TTG CTT AAG TGG AGC TTT GAG T |
| *ZnT10* | CAC CCA GAA TGA GCC AGA AGA C | GAT AAG CGG GAA GGC AGA TGA C |
| *MT-1/2* | forward primer No.1:  GCA CTT CGT GCA AGA AAA GCT  forward primer No.2:  GCA CCT CCT GCA AGA AGA GCT, | reverse primer No.1:  GCA GCC TTG GGC ACA CTT  reverse primer No.2:  GCA GCC CTG GGC ACA CTT |
| *GAPDH* | GAA GGT GAA GGT CGG AGT C | GAA GAT GGT GAT GGG ATT TC |
