# Supplementary material for: ZIP10 as a potential therapeutic target in acute myeloid leukaemia
Source: Br J Haematol. 2025 Jun 30;207(3):767–79. doi: 10.1111/bjh.20229 (PMC12436223; doi:10.1111/bjh.20229)
Supplement: Supplementary file 13 — Appendix S1. [file BJH-207-767-s009.docx]

**Supplementary materials and methods**

**Study design and patient characteristics**

Patients with first diagnosis of AML or disease relapse were recruited from the University RWTH Aachen from 2019 to 2022. Biomaterial from 43 consenting patients (19 female and 24 male, age 29-80, mean age of 64) was archived. Samples were stored at the RWTH centralized Biomaterial Bank (RWTH cBMB) of the Medical Faculty of RWTH Aachen University. The protocol was approved by the ethics committee of the University Hospital RWTH Aachen (Germany) (EK 206/09; project #11-2022) and experiments were conducted in accordance with the ethical standards issued in the Declaration of Helsinki. Inclusion criteria were age >18 years, reconnaissance capability and the successful collection of biomaterials. All AML FAB subtypes were included (1). The patients’ mutational status was analyzed by clinical next generation sequencing (NGS). The respective patient samples were used in different experiments (**Supplementary Table 1**).

**Blood and bone marrow sample acquisition**

Whole blood from bone marrow and peripheral blood was collected using heparin-containing tubes (S-Monovette® Ammonium heparin (02.1064), Sarstedt, Nümbrecht, Germany). Serum tubes with clotting activator (S-Monovette® Serum (02.1063), Sarstedt, Nümbrecht, Germany) were used to collect bone marrow and peripheral blood serum. For the isolation of leukocytes and primary blood mononuclear cells (PBMCs), hydroxyethyl starch (HES) and Pancoll (Pan Biotech, Aidenbach, Germany) were used respectively. For granulocyte isolation, 66% Percoll (GE healthcare, Freiburg, Germany) was used as previously published (2). Adherence to TC-treated dishes (Cellstar®, Greiner Bio-One GmbH, Frickenhausen, Germany) was used to enrich monocytes from PBMCs (3). Western Blot samples (2-5 x 10^6^ cells per vial), trizol samples (2-5 x 10^6^ cells per vial), cell lysates (5 x 10^6^ cells per vial), and serum were stored at -80°C. Living cells (2-5 x 10^6^ cells per vial) were stored at -120°C.

**Atomic Absorption Spectrometry (AAS)**

The total amount of zinc in serum was quantified by flame atomic absorption spectrometry (AAS) using an AAnalyst 800 (Perkin-Elmer, Rodgau, Germany).

**Inductively Coupled Plasma Mass Spectrometry (ICP-MS)**

Total zinc concentration of HNO_3_-digested cells (5 x 10^6^ cells) was determined using an ICP-MS (Agilent 8900 ICP-QQQ, Agilent Technologies, Waldbronn, Germany). Zinc concentration was then normalized to the respective protein concentration of the sample.

**Cultivation of cells, MTT Assays, CFU Assays**

MV4-11, THP-1, NB4, Raji cells and primary cells were cultured in RPMI 1640 (P04-18047; PAN-Biotech, Aidenbach, Germany) with 10% fetal calf serum (FCS) (FBS-12A; Capricorn, Ebsdorfergrund, Germany) and 1% streptomycin/penicillin (Sigma-Aldrich, Steinheim, Germany). NB4 cells were supplemented with 1 % sodium pyruvate (Sigma-Aldrich, Steinheim, Germany). Cell counts were obtained using a CASY cell counter (OMNI Life Science, Bremen, Germany) or a Buerker chamber. During experiments, cell viability was evaluated using propidium iodide (PI) (Life Technologies, Darmstadt, Germany). Treatment was conducted with zinc sulfate (Sigma-Aldrich, Steinheim, Germany) or N,N,N′,N′-tetrakis(2-pyridylmethyl)ethane-1,2-diamine (TPEN) (Sigma-Aldrich, Steinheim, Germany) with the indicated concentrations. Metabolic activity was investigated using 3-(4,5-dimethylthiazol-2-yl)-2,5-diphenyltetrazolium bromide (MTT) assays in a 96 well plate (Greiner Bio-One, Frickenhausen, Germany) as described previously (4). P-nitrophenyl phosphate (pNPP) assay was performed to determine phosphatase activity (5). Telomerase activity was evaluated by using a TRAPeze telomerase detection kit (Sigma-Aldrich, Steinheim, Germany) according to the manufacturer's instructions. To further study cell activation and cell signaling, cells were stimulated with granulocyte colony stimulating factor (G-CSF) (Chugai Pharma, Frankfurt Main, Germany).

Colony forming unit (CFU) assays were carried out by seeding unfractionated bone marrow cells (dependent on blast count) in MethoCult^TM^ (Stem Cell Technologies, Cologne, Germany) supplemented with 20% Iscove’s Modified Dulbecco’s Medium (IMDM) (Thermo Fisher Scientific), 50 ng/ml human stem cell factor (hSCF), 10 ng/ml human interleukin (hIL)-3, 10 ng/ml human granulocyte-macrophage colony-stimulating factor (hGM-CSF), and 3 U/ml human erythropoietin (hEPO) (all ImmunoTools, Friesoythe, Germany) in a cell density of 5,000 - 50,000 cells/ml and 0.5% ciprofloxacin (Fresenius Kabi, Bad Homburg, Germany). Prior to the CFU assay, cells were cultured in Chelex or RPMI1640 medium supplemented with 20 ng/ml hIL-3, 20 ng/ml hIL-6, 50 ng/ml human Fms related receptor tyrosine kinase 3 ligand (hFLT3L), 100 ng/ml hSCF, and 50 ng/ml human thrombopoietin (hTPO) with or without 4 µM TPEN. After 72h of treatment, cell viability was evaluated by using PI and viable cells were applied into the semi-solid medium. Colonies were counted after 14 days.

**Zinc-depleted medium**

Zinc-deficient medium (ZDM) was obtained by treatment of RPMI 1640 (10% FCS and 1% streptomycin/penicillin) with Chelex 100 sodium form ion exchanger (Sigma-Aldrich, Steinheim, Germany) for one hour at 20°C. The Chelex-treated medium was subsequently reconstituted with 500 μM CaCl_2_ (Sigma-Aldrich, Steinheim, Germany) and 400 μM MgCl_2_ (Merck, Darmstadt, Germany) as previously described (6). The medium was controlled for pH. Efficiency of zinc chelation was tested by measuring zinc levels in the medium via AAS. Medium zinc amount of RPMI 1640 medium (10% FCS and 1% streptomycin/penicillin) was 0.194 mg/l (range: 0.174 mg/l – 0.210 mg/l) compared to 0.055 mg/l zinc (range: 0.015 mg/l – 0.089 mg/l) in Chelex-treated medium (data not shown, p<0.0001).

**Measurement of intracellular free zinc and flow cytometry**

To quantify free intracellular zinc, the zinc probe Zinpyr-1 (Santa Cruz Biotechnology, Dallas, USA) was used as previously described (7). Staining was performed with 10 μM Zinpyr-1 for 30 min. Fluorescence was measured via flow cytometry using a Gallios (Beckman Coulter, Brea, USA), Canto or Accuri C6 (both from BD Biosciences, Franklin Lakes, USA). After Fc-receptor block via fetal calf serum, ZIP10 surface staining was done by incubating 1-2 x 10^6^ cells for one hour with at least 20 μg/mL of a commercially available ZIP10 antibody (rabbit polyclonal IgG) (PRS-6099; Biozol, Eching, Germany). Staining was performed using a secondary antibody (anti-rabbit, PE or FITC; Beckman Coulter, Brea, USA). Additional surface markers were stained with antibodies targeting CD34 (FITC or PerCP) and CD3 (PE or APC; BD Biosciences, Franklin Lakes, USA). The corresponding antibody isotypes were used for control stainings.

**mRNA-expression of zinc transporters**

For samples stored in Trizol (Invitrogen, Waltham, USA), mRNA isolation was done via chloroform separation, isopropanol precipitation and ethanol washing. mRNA isolation from whole blood was done using the QIAmp RNA Blood Mini Kit (Quiagen, Hilden, Germany) with subsequent cDNA synthesis (Quiagen, Hilden, Germany). Quantitative analysis of gene expression was performed with fluorescent SYBR green reagent (Applied Biosystems, Waltham, USA) on a 7500 Fast Real-Time PCR System (version 2.3) (Applied Biosystems, Waltham, USA). For most primers, single holding time was 95°C for 10 min, followed by 40 consistent cycles of denaturation at 95°C for 15s and annealing at 60°C for 30s (exception: *ZIP13* (annealing at 62°C) and *ZnT10* (annealing at 65°C)) (8). Primer sequences for *ZIP1*-*ZIP14*, *ZnT1*-*ZnT10*, *MT-1/2* and *GAPDH* are listed in the supplementary material (**Supplementary Table 2**). All samples were run in duplicate. Gene expression is represented as % of GAPDH.

**Western blots**

Samples were centrifuged and pellets from 2-5 x 10^6^ cells were lysed in 100 µL buffer (65 mM Tris-HCl, 2% SDS, 0.01% bromophenol blue, 1% β-mercaptoethanoland 1 mM sodium orthovanadate (all from Sigma-Aldrich, Steinheim, Germany) and 25% glycerol (Fisher Scientific, Hampton, USA). Samples were subsequently sonicated for ten seconds and heated for three to five minutes at 95°C. Protein concentrations were measured by Bradford protein assay. 20 µg protein were used per sample. Following gel separation, proteins were transferred to a nitrocellulose membrane (Bio-Rad, Hercules, USA). Membranes were blocked for one hour with Tris-buffered saline (TBS-T) (containing 20 mM Tris-HCl, 136 mM NaCl (AppliChem, Darmstadt, Germany) and 0.1% Tween 20 (Sigma-Aldrich, Steinheim, Germany)) with 5% milk powder (Saliter, Obergünzburg, Germany). Membranes were incubated overnight at 4°C while shaking with primary antibodies against ZIP10 (rabbit polyclonal IgG) (PRS-6099; Biozol, Eching, Germany), panFlt3 (rabbit polyclonal IgG) (sc-480; Santa Cruz Biotechnology, Dallas, USA), phosphorylated Flt3 (pFlt3) (y589/591) (rabbit monoclonal IgG) (3464S; Cell Signaling Technology, Danvers, USA), panSTAT3 (4904S; Cell Signaling Technology, Danvers, USA) or phosphorylated STAT3 (pSTAT3) (9131S; Cell Signaling Technology, Danvers, USA). Primary antibodies were diluted 1:1000 in TBS-T containing 5% bovine serum albumin (AppliChem, Darmstadt, Germany). After three times washing with TBS-T, membranes were incubated with a secondary antibody (HRP-coupled anti-rabbit IgG; Cell Signaling Technology, Danvers, USA) (1:2000 dilution) for at least four hours at room temperature. Βand visualization was performed using Westar Antares Luminol Enhancer solution (Cynanagen, Bologna, Italy). A LAS-3000 imaging system (Fujifilm Lifescience, Tokyo, Japan) was applied for detecting luminescence with following quantification by using ImageJ software (ImageJ 1.53k; U.S. National Institute of Health, Bethesda, USA).

**ZIP10 antibody production**

The anti-ZIP10 hybridoma (generated by K.M. Taylor, Cardiff University) was cultured in Dulbecco's Modified Eagle Medium (DMEM) low glucose medium supplemented with 100 U/ml penicillin, 100 U/ml streptomycin, 2mM L-glutamine, non-essential amino acids (all from Sigma-Aldrich, Steinheim, Germany) and 15% heat-inactivated “low endotoxin” fetal calf serum (Bio&Sell, Feucht, Germany). Hybridoma cells were cultured in low density and split twice a week. Supernatants were harvested and frozen at -20°C until purification. The antibody is the mouse monoclonal version of a previously published anti-ZIP10B (9).

For antibody purification supernatants were thawed, sterile filtered and diluted 1:1 with binding buffer (1,5 M Glycin/NaOH, 3 M NaCl pH 9,0). A column was loaded with Fast Protein G Sepharose FF Resin (Serva, Heidelberg, Germany). The Sepharose was washed with 7 column volumes distilled water and equilibrated with 4 column volumes binding buffer. Sepharose was loaded with 40 column volumes diluted culture supernatant. Loaded Sepharose was washed with 15 column volumes binding buffer. ZIP10 antibodies were eluted with 2 column volumes elution buffer (0,1M Glycin/HCl pH 2,5) and equilibrated with 1/5 column volume neutralization buffer (1M Tris/HCl pH 9,0). Antibody concentration was determined by Biuret and binding activity of antibodies were checked by surface binding with goat-anti-mouse FITC (Jackson Immunoresearch, West Grove, PA) (**Supplementary Figure 7B-D**).

**Microscopy**

Cultured AML cell lines were stained with 10 μM Zinpyr-1 for 30 min. Afterwards, staining was performed with LysoTracker or MitoTracker (both from Invitrogen, Waltham, USA) according to the manufacturer’s instructions. Stained cells were centrifuged onto microscopic slides by a Cytospin 4 centrifuge (Thermo Scientific, Waltham, USA).

For primary patient material, cells were fixed with 4% paraformaldehyde for ten minutes at room temperature. Cells were then stained overnight with 2.5 μg/mL of a commercial ZIP10 antibody (rabbit polyclonal IgG) (PRS-6099; Biozol, Eching, Germany). After washing with PBS, secondary staining was performed with anti-rabbit-FITC (1:100) for one hour. We used anti-CD34 (ab8536; abcam, Cambridge, UK) (1:50) as an additional primary antibody and subsequent staining with anti-mouse-Cyanin 3 (1:100) for another hour. Imaging was performed with a Leica DM-RXE fluorescence microscope (Leica, Wetzlar, Germany) and a Zeiss Axio Observer Z (Zeiss, Oberkochen, Germany).

**Statistical analysis**

Statistical significances were calculated by using ANOVA in case of multiple comparisons or Student’s t-test when normal distribution was given. Normal distribution was tested with the Kolmogorov-Smirnov and Shapiro-Wilk test. GraphPad Prism software was used for analyses (version 9.1.0; GraphPad Software, Boston, USA). Significance is indicated by *p<0.05, **p<0.01 and ***p<0.001.

**Supplementary Figure 1: Copper level and zinc/copper ratio in patients with first diagnosis or relapse of acute myeloid leukemia**

The protein-adapted cellular copper concentration was measured after cell lysis by using Inductively Coupled Plasma Mass Spectrometry (ICP-MS) in **A)** peripheral blood cells (control subjects: n=6, AML: n=3) and **B)** bone marrow cells (control subjects: n=6, AML: n=8). **C)** Cellular zinc/copper ratio in peripheral blood and **D)** cellular zinc/copper ratio in bone marrow. Data are displayed as mean + standard deviation (SD). Statistical significance was defined as *p<0.05 (Student’s t-test).

**Supplementary Figure 2: mRNA expression of different zinc transporters in the bone marrow of healthy subjects and patients with first diagnosis of acute myeloid leukemia**

mRNA expression in bone marrow cells of *Zrt-, Irt-like Protein* (*ZIP*)*1* (healthy: n=6; AML: n=7; p=0.3760), *ZIP2* (healthy: n=6; AML: n=7; p=0.7343), *ZIP3* (healthy: n=8; AML: n=9; p=0.0834), *ZIP4* (healthy: n=5; AML: n=7; p=0.3868), *ZIP5* (healthy: n=6; AML: n=7; p=0.0891), *ZIP7* (healthy: n=6; AML: n=7; p=0.2325), *ZIP8* (healthy: n=5; AML: n=7; p=0.1499), *ZIP11* (healthy: n=5; AML: n=6; p=0.1685), *ZIP12* (healthy: n=6; AML: n=7), *ZIP13* (healthy: n=5; AML: n=7; p=0.7511), *ZIP14* (healthy: n=8; AML: n=9; p=0.0961), *Zinc Transporter* (*ZnT*)*1* (healthy: n=6; AML: n=7; p=0.3206), *ZnT2* (healthy: n=5; AML: n=7; p=0.6652), *ZnT3* (healthy: n=5; AML: n=7; p=0.5392), *ZnT4* (healthy: n=5; AML: n=7; p=0.4029), *ZnT5* (healthy: n=6; AML: n=7; p=0.3154), *ZnT6* (healthy: n=8 AML: n=9; p=0.2711), *ZnT7* (healthy: n=6; AML: n=7; p=0.6115), *ZnT8* (healthy: n=6; AML: n=7; p=0.1158), *ZnT9* (healthy: n=6; AML: n=7; p=0.2973) and *ZnT10* (healthy: n=5; AML: n=7; p=0.2486). Transporter expression is shown in % expression of the housekeeping gene *GAPDH* + standard deviation (SD). Significance would be indicated by *p<0.05.

**Supplementary Figure 3: mRNA and surface expression of ZIP10**

mRNA expression of all measured bone marrow samples of **A)** *Zrt-, Irt-like Protein* (*ZIP*)*6* (healthy: n=6; AML: n=7; p=0.0429), **B)** *ZIP9* (healthy: n=6; AML: n=7; p=0.0070), **C)** *ZIP10* (=*SLC39A10*) (healthy: n=6; AML: n=7; p=0.0171) and **D)** storage proteins of the *metallothionein family isoform 1 and 2* (*MT-1/2*) (healthy: n=9; AML: n=10; p=0.0253). **E)** Data of *ZIP10* (=*SLC39A10*) mRNA expression from the Leukemia mile study (10–12). Bone marrow from healthy subjects (n=73), AML with normal karyotype (n=351), AML with complex karyotype (n=48), AML with inv(16) (n=28), AML with translocation (15;17) (n=37), AML with translocation (8;21) (n=40) and AML with MLL-rearrangement (n=38). **F)** Data of *ZIP10* (=*SLC39A10*) mRNA expression from the Leukemia mile study (10–12) comparing *ZIP10* expression of bone marrow cells from healthy subjects (n=73) to patients with myelodysplastic neoplasms (n=206).

**Supplementary Figure 4: Effects of zinc deprivation in AML cell lines**

Cell count of **A)** MV4-11 (n=4) and **B)** THP-1 cells (n=4) under zinc-rich (25 μM zinc sulfate, 50 μM zinc sulfate, 100 μM zinc sulfate) and zinc-depleted conditions by using N,N,N′,N′-tetrakis(2-pyridinylmethyl)-1,2-ethanediamine (TPEN) (1 μM TPEN, 2 μM TPEN, 4 μM TPEN) after 72h are indicated. mRNA-expression of *BAK* (**C)** MV4-11 (n=6); **D)** THP-1 (n=3)) after 72h. **E)** + **F)**: Western Blots targeting pSTAT5/panSTAT5 after stimulation of **E)** MV4-11 (n=3, p=0.0033) or **F)** THP-1 cells (n=3, p=0.6957) with 2000 U/ml G-CSF for 45 minutes (“G”) after cultivation in RPMI 1640 or ZDM. **G)** Measurement of phosphatase activity via pNPP assay in MV4-11 (n=3; p=0.5769) and TPH-1 cells (n=12; p=0.0023) after 72h in zinc-deficient medium (ZDM). **H)** Telomerase activity of MV4-11 (n=2) and THP-1 cells (n=3; p=0.8238) after 72h in ZDM. Expression of CD66b in NB-4 cells after ATRA stimulation for 72h in **I)** zinc-rich (50 μM zinc sulfate) (n=9, p=0.0295) or **J)** ZDM (n=8, p=0.0390). One-Way ANOVA - Dunnett’s multiple comparison (**A)** – **D)**) or Student’s t-test were used to calculate statistical significance. Data are displayed as mean + standard deviation (SD). Significance is indicated by *p<0.05 and **p<0.01.

**Supplementary Figure 5: Experimental modulation of zinc levels in cell culture and staining of primary patient material**

**A) + B):** Fold change in free intracellular zinc by using FluoZin3-AM compared to control after 48h incubation of NB-4 cells in **A)** RPMI 1640 plus 50 μM zinc sulfate (p=0.0005) or **B)** ZDM (p<0.0001). **C)** In parallel to experiments focusing on the PML/RARα fusion protein, free intracellular zinc was also stained by using Zinpyr-1 in NB-4 cells after 72h in ZDM (n=7, p=0.0002). **D)** Free intracellular zinc concentration over time in NB-4 cells during differentiation with 1 µM ATRA by using normal RPMI 1640 medium. Zinc amount was measured by using the zinc dye FluoZin3-AM with subsequent calculation of the zinc concentration as previously described (after 24h: n=3, p=7454; after 48h: n=8, p=0.0009; after 72h: n=8, p=0.0049; after 144h: n=7, p=0.0018; One-way ANOVA) (7). **E)** Representative microscopic images that show co-staining of primary AML blasts with Zinpyr-1 (green) and LysoTracker (red) (n=4). **F)** Co-staining of Zinpyr-1 (green) with MitoTracker (red) in primary AML blasts. Student’s t-test was used to calculate statistical significance. Data are displayed as mean + standard deviation (SD). Significance is indicated by **p<0.01 and ***p<0.001.

**Supplementary Figure 6: Experimental analyses of primary patient material**

**A)** Immunohistochemical co-staining of ZIP10 (green), CD34 (red) and DAPI (blue). **B)** Exemplary flow cytometry analysis from one out of four AML patients showing CD34^+^ cells that simultaneously express ZIP10 on the cell surface (n=4). **C) + D)**: Delta between the percentage of CD34^+^ cells that show PI-positivity and CD34^-^ cells (remaining cells) that show PI-positivity after 72h in **C)** 4 μM TPEN (n=4, p=0.0130) or **D)** ZDM (n=3, p=0.0145). **E) + F)**: Percentage of **E)** CD34^+^ cells and **F)** CD3^+^ cells among all cells after 72h incubation in ZDM vs. control medium (RPMI1640). Data are displayed as mean + standard deviation (SD). Significance is indicated by *p<0.05.

**Supplementary Figure 7: Treatment by using a ZIP10 antibody**

**A)** Cell count (n=13) of MV4-11 cells over 72h by using different concentrations of elution buffer (EB) (1x, 2.5x and 3.5x) that correspond to the concentrations used for the treatment with ZIP10, ZIP10 (2.5x) and ZIP10 (3.5x). **B)** Percentage of purified ZIP10 antibody binding to the cell surface of MV4-11 cells (n=11) directly after incubation and after 72h (ZIP10, ZIP10 (2.5x), ZIP10 (3.5x)). Binding of **C)** isotype control and the **D)** purified ZIP10 antibody after 24h incubation with MV4-11 cells. Two-way ANOVA - Dunnett’s multiple comparison (**A)**) or One-way ANOVA - Dunnett’s multiple comparison (**B)**) were used to calculate statistical significance. Data are displayed as mean + standard deviation (SD). Significance is indicated by *p<0.05.

**Supplementary Figure 8: Correlations of ZIP10 and zinc levels with different patient characteristics**



**A)** *ZIP10* expression (in peripheral blood, bone marrow or leukapheresis material) for patients with initial diagnosis of AML (n=450), AML in remission (n=25), residual disease (n=131) or relapsed AML (n=37). Significant differences by using a One-way ANOVA are shown between initial diagnosis vs. remission (p<0.0001), initial diagnosis vs. relapse (p=0.015), remission vs. residual disease (p<0.0001), remission vs. relapse (p<0.0001) and residual disease vs. relapse (p=0.0003). *ZIP10* expression in the **B)** peripheral blood (n=408, p<0.0001) or **C)** bone marrow (n=521, p<0.0001) are shown from patients with acute myeloid leukemia (AML) dependent on the present blast count. **D)** Association between ZIP10 protein detection in the peripheral blood (Western Blot) and the peripheral blood blast count based on our data (n=19, p=0.0165, R^2^=0.29). **E)** Association between the blast percentage in the bone marrow and the measured cellular zinc amount of bone marrow cells based on our data (n=8, p=0.0847, R^2^=0.42). **F)** *ZIP10* expression in healthy mononuclear cells (MNCs) from the bone marrow (n=19) compared to healthy CD34^+^ cells (n=17) (t-test, p<0.0001). Reads per kilobase of exon per million reads mapped (RPKM) was used for normalization of *ZIP10* expression. Data from the BeatAML2.0 study (**A)** - **C)** + **F)**) study (Tyner *et al,* 2018; Bottomly *et al,* 2022) and our study (**D)** + **E)**) were used for analysis. Data are displayed as mean +/- standard deviation (SD). Significance is indicated by *p<0.05, ***p<0.001, and ****p<0.0001.

**Supplementary Figure 9: Correlations of ZIP10 with patient characteristics and outcome**

To further correlate *ZIP10* (=*SLC39A10*) expression with patient characteristics and clinical outcome, public available data from the BeatAML2.0 study (13, 14) were used. Reads per kilobase of exon per million reads mapped (RPKM) was used for normalization of *ZIP10* expression. **A)** *ZIP10* expression dependent on the FAB classification FAB M0 (n=26), M1 (n=68), M2 (n=66), M3 (n=18), M4 (n=65), M5 (n=74), M6 (n=6), M7 (n=3) and NOS (n=5). Statistical differences are shown by using One-way ANOVA as differences between FAB M1 vs. M2 (p<0.0001), FAB M1 vs. M4 (p<0.0001), FAB M1 vs. M5 (p<0.0001) and FAB M1 vs. M7 (p=0.0079). **B)** Initial *ZIP10* expression in all patients with de-novo AML who died (n=80) in relation to the overall survival time (p=0.085). **C)** *ZIP10* expression in patients with relapsed *FLT3^ITD^*-positive AML who died in the course of disease (n=6) in relation to overall survival (p=0.0390). **D)** *ZIP10* low and high mRNA expression in all patients with *FLT3^ITD^*- and *FLT3^TKD^*-positive AML (n=134) in relation to the response duration after induction therapy (p=0.0002; Gehan-Breslow-Wilcoxon test). Significance is indicated by **p<0.01 and ****p<0.0001.

**Supplementary Figure 10: Changes in cellular copper under zinc-deficient conditions**

MV4-11 and THP-1 cells were incubated with zinc (100 μM), TPEN (4 μM), or Chelex-treated media for 72 hours to modulate intracellular metal availability. We then measured total cellular copper and zinc levels in 1,000,000 cells per condition by using Inductively Coupled Plasma Mass Spectrometry (ICP-MS) (n=3). Cellular copper (**A)** MV4-11 + **B)** THP-1) and zinc/copper ratio (**C)** MV4-11 + **D)** THP-1) are shown. One-way ANOVA - Dunnett’s multiple comparison was used to calculate statistical significance. Data are displayed as mean + standard deviation (SD). Statistical significance was defined as *p<0.05.

Literature Cited

1. Bennett JM, Catovsky D, Daniel MT, Flandrin G, Galton DA, Gralnick HR et al. Proposals for the classification of the acute leukaemias. French-American-British (FAB) co-operative group. Br J Haematol 1976; 33(4):451–8. Available from: URL: https://pubmed.ncbi.nlm.nih.gov/188440/.

2. Schröder AK, Ohe M von der, Kolling U, Altstaedt J, Uciechowski P, Fleischer D et al. Polymorphonuclear leucocytes selectively produce anti-inflammatory interleukin-1 receptor antagonist and chemokines, but fail to produce pro-inflammatory mediators. Immunology 2006; 119(3):317–27.

3. Klinder A, Markhoff J, Jonitz-Heincke A, Sterna P, Salamon A, Bader R. Comparison of different cell culture plates for the enrichment of non-adherent human mononuclear cells. Exp Ther Med 2019; 17(3):2004–12.

4. Kessels JE, Wessels I, Haase H, Rink L, Uciechowski P. Influence of DNA-methylation on zinc homeostasis in myeloid cells: Regulation of zinc transporters and zinc binding proteins. J Trace Elem Med Biol 2016; 37:125–33.

5. Haase H, Ober-Blöbaum JL, Engelhardt G, Hebel S, Heit A, Heine H et al. Zinc signals are essential for lipopolysaccharide-induced signal transduction in monocytes. J Immunol 2008; 181(9):6491–502.

6. Dubben S, Hönscheid A, Winkler K, Rink L, Haase H. Cellular zinc homeostasis is a regulator in monocyte differentiation of HL-60 cells by 1 alpha,25-dihydroxyvitamin D3. J Leukoc Biol 2010; 87(5):833–44.

7. Rolles B, Maywald M, Rink L. Intracellular zinc during cell activation and zinc deficiency. J Trace Elem Med Biol 2021; 68:126864.

8. Kloubert V, Wessels I, Wolf J, Blaabjerg K, Janssens V, Hapala J et al. Zinc deficiency leads to reduced interleukin-2 production by active gene silencing due to enhanced CREMα expression in T cells. Clin Nutr 2021; 40(5):3263–78.

9. Nimmanon T, Ziliotto S, Ogle O, Burt A, Gee JMW, Andrews GK et al. The ZIP6/ZIP10 heteromer is essential for the zinc-mediated trigger of mitosis. Cell Mol Life Sci 2021; 78(4):1781–98.

10. Bagger FO, Sasivarevic D, Sohi SH, Laursen LG, Pundhir S, Sønderby CK et al. BloodSpot: a database of gene expression profiles and transcriptional programs for healthy and malignant haematopoiesis. Nucleic Acids Res 2016; 44(D1):D917-24.

11. Haferlach T, Kohlmann A, Wieczorek L, Basso G, Kronnie GT, Béné M-C et al. Clinical utility of microarray-based gene expression profiling in the diagnosis and subclassification of leukemia: report from the International Microarray Innovations in Leukemia Study Group. J Clin Oncol 2010; 28(15):2529–37.

12. Verhaak RGW, Wouters BJ, Erpelinck CAJ, Abbas S, Beverloo HB, Lugthart S et al. Prediction of molecular subtypes in acute myeloid leukemia based on gene expression profiling. haematol 2009; 94(1):131–4.

13. Bottomly D, Long N, Schultz AR, Kurtz SE, Tognon CE, Johnson K et al. Integrative analysis of drug response and clinical outcome in acute myeloid leukemia. Cancer Cell 2022; 40(8):850-864.e9.

14. Tyner JW, Tognon CE, Bottomly D, Wilmot B, Kurtz SE, Savage SL et al. Functional genomic landscape of acute myeloid leukaemia. Nature 2018; 562(7728):526–31.
