# Supplementary material for: ZIP10 as a potential therapeutic target in acute myeloid leukaemia
Source: Br J Haematol. 2025 Jun 30;207(3):767–79. doi: 10.1111/bjh.20229 (PMC12436223; doi:10.1111/bjh.20229)
Supplement: Supplementary file 14 — Appendix S2. [file BJH-207-767-s002.docx]

**Supplementary Discussion:**

To maintain the flow of the main manuscript and adhere to word count limitations, we have moved certain aspects of the discussion to the supplementary material. This allows for a more in-depth exploration of key findings without disrupting the core narrative. The increase in free intracellular zinc, as observed in our study, is of particular interest since this compartment is most relevant for zinc-mediated cell signaling (1). It was recently shown that free intracellular zinc participates in a high number of different signaling cascades in immune cells (2). Concordant to these observations, we found upregulated zinc influx transporters, ZIPs, that mechanistically explain higher cytosolic zinc levels in AML cells (**Figure 2**). In addition, data from the Leukemia Mile Study (n=542) (3–5) and BeatAML2.0 study (n=805) (6, 7) showed that *ZIP10* was overexpressed in all analyzed AML subtypes. Consistent with our findings, previous studies suggest that the ZIP10/ZIP6 heterodimer may facilitate cell proliferation (8, 9). However, the respective mRNA-expression of *MT-1/2* was decreased at the same time. MT proteins, which are important for bivalent trace element storage, are able to protect cells from zinc-induced toxicity (10). Considering that malignant cells showed increased *ZIP* expression, but decreased expression of storage proteins like *MT-1/2*, strengthened our hypothesis that increased free intracellular zinc may be an important pathogenic factor of AML blasts.

In both MV4-11 and THP-1 cells, zinc deficiency was found to reduce phosphorylation of STAT3 and FLT3, suggesting a potential mechanism through which zinc influences cellular signaling. This reduction is partly explained by increased phosphatase activity in THP-1 cells, a phenomenon also previously reported in lymphocytes (11). In MV4-11 cells, a *FLT3^ITD^*-mutated AML cell line, decreased phosphatase activity was not observed. However, reduced STAT5 phosphorylation was detected, suggesting that ZDM may influence cell signaling pathways through multiple mechanisms, warranting further investigation.

Previous studies have demonstrated that zinc deprivation can promote cell differentiation in monocytes (12, 13), which is particularly relevant given that many AML subtypes exhibit monocyte-like morphology (14). Consistent with these findings, we observed similar results using the NB-4 promyelocytic leukemia cell line. Zinc supplementation decreased ATRA-induced differentiation of NB-4 after three and five days. This supports the observation that higher zinc levels can be observed in some patients with MDS, a disease that is characterized by dysplastic BM cells with decreased cell differentiation (15). Zinc deficiency, on the other hand, increased the rate of NB-4 cell differentiation due to the degradation of the PML-RARα fusion protein (16).

Patients who entered remission after AML diagnosis showed significantly decreased *ZIP10* expression (**Supplementary Figure 8A**). Furthermore, patients with AML relapse had significantly higher *ZIP10* mRNA levels compared to patients with initial diagnosis (**Supplementary Figure 8A**). As such, *ZIP10* mRNA expression showed a linear correlation with blast count in the PB (**Supplementary Figure 8B**) as well as BM (**Supplementary Figure 8C**). In our AML cohort, we observed similar results for ZIP10 protein levels in PB (**Supplementary Figure 8D**, p=0.0165) and cellular zinc content in BM (**Supplementary Figure 8E**, p=0.0847). Accordingly, we showed that CD34^+^ AML blasts had a strong expression of the ZIP10 surface protein.

Nevertheless, *ZIP10* is not only expressed in malignant AML blasts but might be a transport protein that supports the function of monocytes and CD34^+^ hematopoietic stem cells (HSCs). Data from the AMLBeat2.0 study (6, 7) confirms that healthy CD34^+^ cells express significantly more *ZIP10* than other blood cells (**Supplementary Figure 8F**). Strikingly, AML with reduced/minimal differentiation (FAB classification) showed highest expression of *ZIP10* (**Supplementary Figure 9A**).

Primary CD34^+^ AML cells showed higher free intracellular zinc compared to CD34^-^ cells and demonstrated more zinc uptake *in vitro* reinforcing the hypothesis that increased intracellular zinc levels may be important for AML cell survival. In lymphocytes, zinc is released from lysosomal stores during cell activation, highlighting its role as a second messenger (17, 1, 18). Our findings further show that in AML blasts, the zinc-specific dye Zinpyr-1 co-localizes with lysosomes, suggesting a similar regulatory role of lysosomal zinc in AML.

Especially in CD34^+^ cells we found increased cell death caused by zinc-deprivation. In ZDM, we saw a decreased percentage of CD34^+^ cells, but no significant change in CD3^+^ cells indicating a more pronounced toxic effect of zinc depletion on malignant cells compared to healthy BM cells. This conclusion is further supported by our CFU assay results, which demonstrate a reduced number of colonies following cultivation in zinc-depleted medium. This suggests that leukemic stem cells (LSCs) may be more susceptible to zinc deprivation.

Finally, we performed a co-expression analysis and observed that the *FLT3* gene shows a positive correlation with *ZIP10* (=*SLC39A10*) expression. This is particularly interesting as aberrations in *FLT3* are associated with increased cell proliferation in AML (19) and unfavorable prognosis (20). In addition, it should be noted that the vast majority of AML cell lines showed a very strong dependency on the ZIP10 molecule.

Interestingly, high *ZIP10* expression tended to be associated with decreased overall survival in patients with de-novo AML (**Supplementary Figure 9B**, n=80, p=0.0855) and relapsed *FLT3^ITD^*-mutant patients (**Supplementary Figure 9C**, n=6, p=0.0390) who died from the disease. In patients with *FLT3^ITD^* or *FLT3^TKD^* for whom the response duration after induction therapy was recorded, we found that high *ZIP10* expression (by using the median as a cut-off) was related with shortened duration of therapy response (**Supplementary Figure 9D**, n=134, p=0.0002 (Gehan-Breslow-Wilcoxon test)). Although low zinc levels during our experiments may influence intracellular copper concentrations and thus potentially contribute to cuproptosis (21), this mechanism appears unlikely. We observed no significant differences in copper levels between primary control and AML cells (**Supplementary Figure 1**), nor any substantial increase in cellular copper during cell culture in ZDM (**Supplementary Figure 10**).

Literature Cited

1. Rink L, Gabriel P. Zinc and the immune system. Proc Nutr Soc 2000; 59(4):541–52.

2. Rolles B, Maywald M, Rink L. Intracellular zinc during cell activation and zinc deficiency. J Trace Elem Med Biol 2021; 68:126864.

3. Bagger FO, Sasivarevic D, Sohi SH, Laursen LG, Pundhir S, Sønderby CK et al. BloodSpot: a database of gene expression profiles and transcriptional programs for healthy and malignant haematopoiesis. Nucleic Acids Res 2016; 44(D1):D917-24.

4. Haferlach T, Kohlmann A, Wieczorek L, Basso G, Kronnie GT, Béné M-C et al. Clinical utility of microarray-based gene expression profiling in the diagnosis and subclassification of leukemia: report from the International Microarray Innovations in Leukemia Study Group. J Clin Oncol 2010; 28(15):2529–37.

5. Verhaak RGW, Wouters BJ, Erpelinck CAJ, Abbas S, Beverloo HB, Lugthart S et al. Prediction of molecular subtypes in acute myeloid leukemia based on gene expression profiling. haematol 2009; 94(1):131–4.

6. Bottomly D, Long N, Schultz AR, Kurtz SE, Tognon CE, Johnson K et al. Integrative analysis of drug response and clinical outcome in acute myeloid leukemia. Cancer Cell 2022; 40(8):850-864.e9.

7. Tyner JW, Tognon CE, Bottomly D, Wilmot B, Kurtz SE, Savage SL et al. Functional genomic landscape of acute myeloid leukaemia. Nature 2018; 562(7728):526–31.

8. Nimmanon T, Ziliotto S, Ogle O, Burt A, Gee JMW, Andrews GK et al. The ZIP6/ZIP10 heteromer is essential for the zinc-mediated trigger of mitosis. Cell Mol Life Sci 2021; 78(4):1781–98.

9. Li H, Shen X, Ma M, Liu W, Yang W, Wang P et al. ZIP10 drives osteosarcoma proliferation and chemoresistance through ITGA10-mediated activation of the PI3K/AKT pathway. J Exp Clin Cancer Res 2021; 40(1):340. Available from: URL: https://jeccr.biomedcentral.com/articles/10.1186/s13046-021-02146-8#citeas.

10. Coyle P, Philcox JC, Carey LC, Rofe AM. Metallothionein: the multipurpose protein. Cell Mol Life Sci 2002; 59(4):627–47.

11. Plum LM, Brieger A, Engelhardt G, Hebel S, Nessel A, Arlt M et al. PTEN-inhibition by zinc ions augments interleukin-2-mediated Akt phosphorylation. Metallomics : integrated biometal science 2014; 6(7):1277–87. Available from: URL: https://pubmed.ncbi.nlm.nih.gov/24759986/.

12. Dubben S, Hönscheid A, Winkler K, Rink L, Haase H. Cellular zinc homeostasis is a regulator in monocyte differentiation of HL-60 cells by 1 alpha,25-dihydroxyvitamin D3. J Leukoc Biol 2010; 87(5):833–44.

13. Kim B, Kim HY, Yoon BR, Yeo J, Jung J in, Yu K-S et al. Cytoplasmic zinc promotes IL-1β production by monocytes and macrophages through mTORC1-induced glycolysis in rheumatoid arthritis. Sci Signal 2022; 15(716):eabi7400.

14. Aurelius J, Thorén FB, Akhiani AA, Brune M, Palmqvist L, Hansson M et al. Monocytic AML cells inactivate antileukemic lymphocytes: role of NADPH oxidase/gp91(phox) expression and the PARP-1/PAR pathway of apoptosis. Blood 2012; 119(24):5832–7.

15. Siddiqui S, Ramlal R. "Myelodysplasia" from copper deficiency. Blood 2019; 133(8):883. Available from: URL: https://ashpublications.org/blood/article/133/8/883/260587/Myelodysplasia-from-copper-deficiency.

16. Görg R, Büttgenbach A, Jakobs J, Kurtoğlu Babayev FH, Rolles B, Rink L et al. Leukemia cells accumulate zinc for oncofusion protein stabilization. J Nutr Biochem 2024; 123:109482.

17. Wessels I, Fischer HJ, Rink L. Dietary and Physiological Effects of Zinc on the Immune System. Annu Rev Nutr 2021; 41:133–75.

18. Maywald M, Rink L. Zinc in Human Health and Infectious Diseases. Biomolecules 2022; 12(12):1748.

19. Nogami A, Watanabe D, Okada K, Akiyama H, Umezawa Y, Nagao T et al. FLT3-ITD Enhances Proliferation and Survival of AML Cells through Activation of RSK1 to Upregulate the mTORC1/eIF4F Pathway Cooperatively with PIM or PI3K and to Inhibit Bad and Bim. Blood 2019; 134(Supplement_1):1425. Available from: URL: https://ashpublications.org/blood/article/134/Supplement_1/1425/427487/FLT3-ITD-Enhances-Proliferation-and-Survival-of.

20. Daver N, Venugopal S, Ravandi F. FLT3 mutated acute myeloid leukemia: 2021 treatment algorithm. Blood Cancer J 2021; 11(5):104.

21. Tang D, Kroemer G, Kang R. Targeting cuproplasia and cuproptosis in cancer. Nat Rev Clin Oncol 2024; 21(5):370–88. Available from: URL: https://www.nature.com/articles/s41571-024-00876-0.
